# Amino acid–dependent regulation of insulin-like peptide signaling is mediated by TOR and GATA factors in the disease vector mosquito *Aedes aegypti*

**DOI:** 10.1073/pnas.2303234120

**Published:** 2023-08-14

**Authors:** Lin Ling, Alexander S. Raikhel

**Affiliations:** ^a^School of Life Science and Technology, Key Laboratory of Developmental Genes and Human Disease, Southeast University, Nanjing 210096, China; ^b^Department of Entomology, University of California, Riverside, CA 92521; ^c^Institute of Integrative Genome Biology, University of California, Riverside, CA 92521

**Keywords:** mosquito, insulin, TOR, GATA, CRISPR-Cas9

## Abstract

*Aedes aegypti* female mosquitoes require vertebrate blood for their egg production and consequently they become vectors of devastating human diseases. Amino acids (AAs) and nutrients originating from a blood meal activate vitellogenesis and fuel embryo development of anautogenous mosquitoes. Insulin-like peptides (ILPs) are indispensable in reproducing female mosquitoes, regulating glycogen and lipid metabolism, and other essential functions. However, how ILPs coordinate their action in response to the AA influx in mosquito reproduction was unknown. We report here that the AA/Target of Rapamycin (TOR) signaling pathway regulates ILPs through GATA transcription factors (TFs). AA infusion combined with RNA-interference TOR silencing of revealed their differential action on ILPs, elevating circulating levels of several ILPs but inhibiting others, in the female mosquito. Experiments involving isoform-specific CRISPR-Cas9 genomic editing and chromatin immunoprecipitation assays showed that the expression of *ilp4ilp6,* and *ilp7* genes was inhibited by the GATA repressor (GATAr) isoform in response to low AA-TOR signaling, while the expression of *ilp1ilp2ilp3ilp5,* and *ilp8* genes was activated by the GATA activator isoform after a blood meal in response to the increased AA-TOR signaling. FoxO, a downstream TF in the insulin pathway, was involved in the TOR-GATAr-mediated repression of *ilp4ilp6,* and *ilp7* genes. This work uncovered how AA/TOR signaling controls the ILP pathway in modulation of metabolic requirements of reproducing female mosquitoes.

## Significance

*Aedes aegypti* female mosquitoes are vectors of human viral diseases because they require vertebrate blood for their reproduction. Using CRISPR-Cas9 gene modification, transgenesis, and RNA interference in the mosquito, we identified the molecular mechanism underlying the nutritional pathway, in which the serine/tyrosine kinase target of rapamycin (TOR) mediates the amino acid (AA) signaling through GATA transcription factors and affects insulin-like peptides (ILP). GATA repressor (GATAr) inhibits the transcription of *ilp4ilp6*, and *ilp7* genes. FoxO, a downstream TF in the insulin pathway, prevents the TOR-GATAr-mediated repression of these *ilp* genes. After the blood meal–mediated AA influx, GATA activator elevates the expression of *ilp1ilp2ilp3ilp5*, and *ilp8* genes. Targeting AA-dependent reproductive processes may provide effective means in controlling mosquito population.

Hematophagous female mosquitoes are important vectors of numerous devastating human diseases (1). Mosquito-borne diseases are among the world’s leading causes of death and illness today, and mosquito control is of the utmost importance for public health (2–4). The distribution of mosquito-borne diseases is determined by a complex set of social, demographic, and environmental factors. The *Aedes aegypti* mosquito is the major vector of Dengue fever, yellow fever, Chikungunya, Zika, and other vector-borne viral human diseases (5–9). Every female mosquito produces a batch of about 120 eggs at one time, and three or more batches in its lifespan, mounting an impressive reproductive capacity. Each reproductive cycle of a female mosquito consists of two phases: a previtellogenic phase (posteclosion; PE), during which female’s tissues are preparing for egg development, and a vitellogenic phase (post blood meal; PBM), during which egg development occurs following blood feeding. The first phase lasts about 3 to 4 d, during which a female feed on nectar until it finds a host. The second, the vitellogenic phase, is initiated following blood feeding and usually lasts 72 h or until a female finds another host and feeds on blood to initiate another gonadotrophic cycle. Thus, the two phases of each gonadotropic cycle are drastically different in dietary intake—nectar feeding (carbohydrate) during the PE and blood feeding (protein) during PBM (10–12). Feeding on carbohydrate during the PE phase facilitates mating, flight, host seeking, and preparation for egg production (11). Elucidation of the molecular mechanisms underlying nutrient and metabolic needs in support of female mosquito reproduction is essential for implementation of vector population-control approaches.

Insulin is the major hormone regulating nutrition activating the uptake of glucose, fatty acids, and amino acids (AAs) along with their storage in the form of glycogen, lipid, and protein. Dysregulation of the insulin pathway leads diabetes and other metabolic disorders in humans (13, 14). Insect insulin-like peptides (ILPs) are 6- to 8-kDa molecules with the insulin fold motif that belong to the insulin superfamily and are encoded by multiple genes (15, 16). ILPs are produced by the insect brain and other tissues and function in the coordination of metabolism, development, and reproduction (16, 17). Insects store glycogen and triacylglycerides (TAGs) as energy reserves in the adipocytes of the fat body for growth and reproduction. There are eight different *ilps* genes (*ilps* 1-8) in the genome of the *Ae. aegypti* mosquito. Previous studies have revealed that CRISPR-Cas9 disruption of each ILP in female mosquitoes caused differential degrees of deficiency in growth, nutrient storage, carbohydrate/lipid balance, and egg development (18, 19).

AAs from a blood meal are necessary for anautogenous female mosquitoes to complete a gonotrophic cycle (10). The level of free AAs rises sharply in the mosquito hemolymph as digestion of the vertebrate blood meal occurs in the midgut (20). AAs are absorbed and carried to the mosquito fat body cells by specific transporters (21, 22). The fat body, an insect organ analogous to the vertebrate liver and adipose tissue combined, serves as the nexus for nutrient sensing, lipid storage, and endocrine signaling to the brain and reproductive organs (23, 24). AAs provide building blocks for protein synthesis and are also used for energy expenditure; in addition, free AAs are known as regulators of cellular signaling to coordinate metabolism (20, 25). However, how insulin signals coordinate their action with the AA signaling during mosquito reproduction remains largely unknown. This knowledge is vital for a thorough understanding of the mechanisms controlling anautogenous reproduction in mosquitoes and may provide the background for the utilization of these peptide hormones or their regulators for management of mosquitoes and vector-borne diseases.

The ILP and the target of rapamycin (TOR) pathways are two main nutrient sensors (26–28). AAs are critical signals for TOR activation (29). In the mosquito, after receiving the nutrition signal from AA-TOR, the activated GATAa (GATA activator) displaces the GATAr (GATA repressor) isoform to enhance gene expression (30). GATAr or GATAa knockouts in mosquitoes are lacking because of challenges related to alternative splicing of the GATA gene pre-mRNA. We examined the role of TOR-mediated AA signaling in *ilp* expression by comparing the analysis of AA infusion, RNA-interference (RNAi) silencing of TOR, and isoform-specific CRISPR-Cas9 genomic editing of GATAr or GATAa. We found that TOR differentially regulates *ilp* expression in female mosquitoes. Further, we showed that the expression of *ilp4*, *ilp6*, and *ilp7* was inhibited by the GATAr isoform in response to low AA-TOR signaling, while the expression of *ilp1*, *ilp2*, *ilp3*, *ilp5*, and *ilp8* was activated by the GATAa isoform in response to the increased AA-TOR signaling. Importantly, FoxO, a downstream transcription factor (TF) in the insulin signaling pathway, is involved as an interrupter role in TOR-GATAr-mediated repression of *ilp4*, *ilp6*, and *ilp7* expression. Thus, our results reveal a signaling mechanism of how ILPs are controlled to coordinate their actions with the nutrient availability and reproductive needs in mosquitoes.

## Results

### Differential Actions of AA and TOR on *ilp* Gene Expression.

**Fig. 1.**
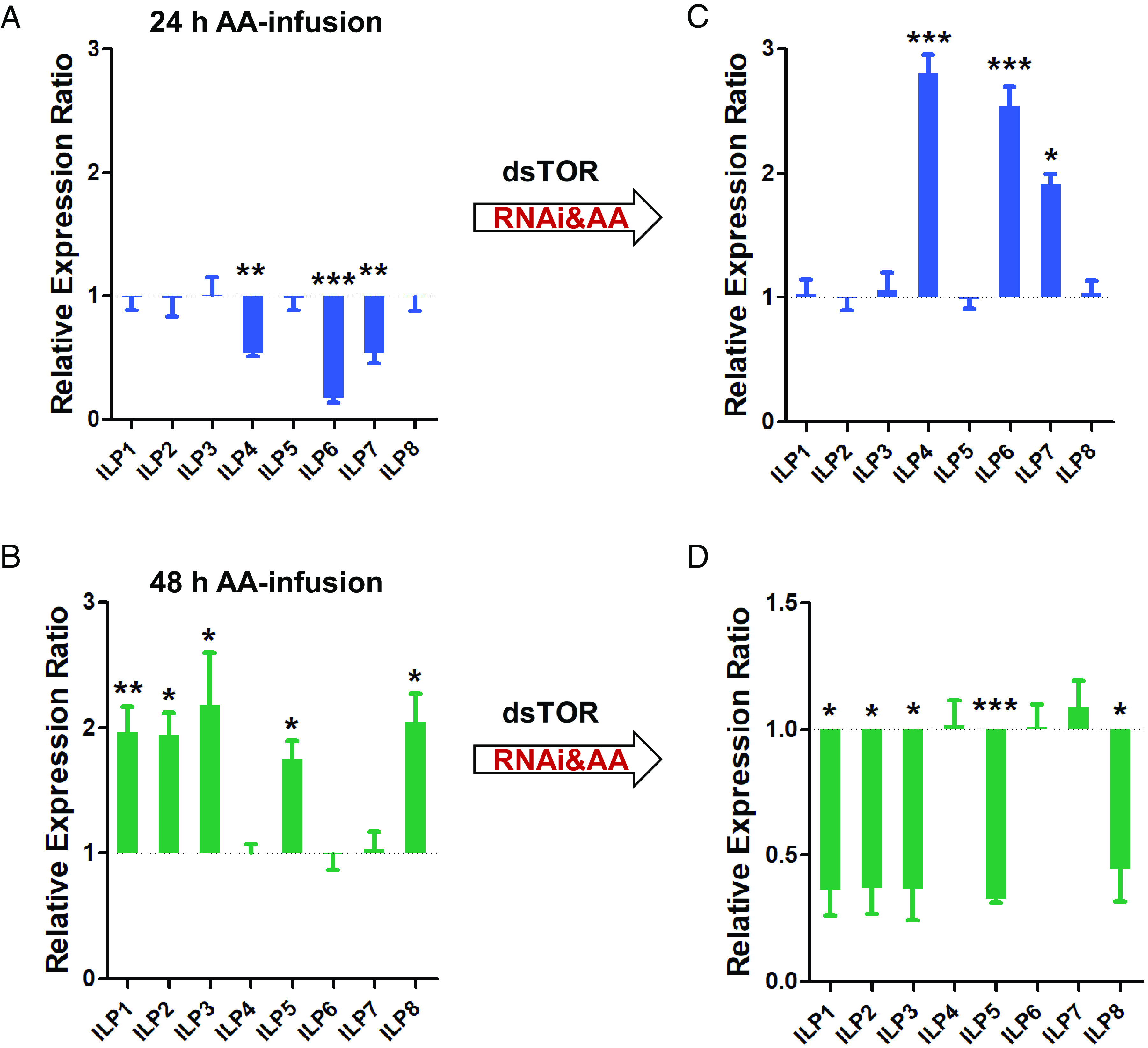
Comparative analysis of *ilp* transcript abundance in AA-infused and *dsTOR* RNAi female mosquitoes. (*A*) The infusion of AAs for 24 h (0.083 μL/h) repressed *ilp-4*, -*6*, and -*7* transcripts but not the other *ilps*. (*B*) The 48-h AA infusion caused no change in *ilp*-*4*, -*6*, and -*7* transcripts but activated the other five (*ilps 1235*, and *8*). (*C*) RNAi knockdown of *TOR* combining 24-h AA infusion elevated the AA-down-regulated (*ilps 46*, and *7*) *ilp* transcripts. (*D*) RNAi knockdowns of *TOR* combining 48-h AA infusion reduced the AA-up-regulated (*ilps 1235*, and *8*) *ilp* transcripts. *dsLuc* (RNAi-*luciferase*) and infusion of saline solution were used as controls. Data represent three biological replicates with 30 individuals in each and are shown as mean ± SEM; *P < 0.05, **P < 0.01, ***P < 0.001.

Our previous study has demonstrated that *ilps 4*, *6*, and 7 are up-regulated during the PE phase and down-regulated after blood feeding. In contrast, *ilps 1*, *2*, *3*, *5*, and *8* exhibited high levels of expression during the PBM phase (*SI Appendix*, Fig. S1) (31). Thus, in addition to hormonal input, identified in the abovementioned work, a direct nutritional signaling maybe involved in controlling *ilp* gene expression. Signaling through the TOR pathway is the key to activation of vitellogenesis. The AA transporter Slimfast is critical for this signaling (10, 21, 32). To examine whether AA signaling is engaged in regulating *ilp* gene expression, we first conducted AA infusion into the hemocoel of the adult female mosquito during the PE phase. It has been shown that the addition of a balanced AA mixture into the hemocoel of previtellogenic mosquitoes stimulates oogenesis (33). We introduced the solution containing 17 AAs into 3-d-old female *A. aegypti* adults for 24 h (0.083 μL/h × 24 h = 2.0 μL/24 h) or 48 h (4.0 μL/48 h) using the infusion method reported before (33) (*SI Appendix*, Fig. S2 *A* and *B*). The transcript levels of each of the eight *ilps* were measured using real-time quantitative PCR (RT-qPCR) analysis. After the 24-h AA infusion *ilps 4*, *6*, and *7* were down-regulated, while no effect was noticed on the expression of *ilps 1*, *2*, *3*, *5*, and *8*. This indicates that although the low level of AAs was sufficient for reducing the expression level of *ilps* in the first group, it was not sufficient for *ilp* activation of the second group (Fig. 1*A*). However, these five *ilps* (*1*, *2*, *3*, *5*, and *8*) were significantly up-regulated in mosquitoes infused with AAs for 48 h (Fig. 1*B*). Control females infused with a saline solution exhibited the same pattern of *ilp* gene expression as untreated mosquitoes. Thus, the AA signaling plays a critical role in regulating *ilp* gene expression in reproducing female mosquitoes.

To understand whether TOR is involved in mediating the AA signaling in coordinating *ilp* expression, we conducted TOR RNAi in a combination of AA infusion. Female mosquitoes were injected with double-stranded TOR RNA (ds*TOR*) or ds*Luciferase* (ds*Luc*) as a control at 6 h PE after adult emergence (*SI Appendix*, Fig. S2*C*). After 3 d of recovery, the dsRNA-injected females were infused with AAs for 24 h or 48 h (0.083 μL/h). The fat body tissue was dissected and used for RT-qPCR analysis. The transcript levels of AA-down-regulated *ilp* genes (*ilps 4, 6*, and *7*) increased in *dsTOR* RNAi mosquitoes infused for 24 h, while no response was observed for the AA-up-regulated *ilp* genes (*ilps 1, 2, 3, 5*, and *8*) (Fig. 1*C*). After 48-h infusion, no response was detected for AA-down-regulated *ilp* genes (*ilps 4, 6,* and *7*) in *dsTOR* RNAi mosquitoes, while the AA-up-regulated *ilp* genes (*ilps 1, 2, 3, 5*, and *8*) were down-regulated (Fig. 1*D*). These results clearly indicate that TOR is requited for mediating the AA signaling. Moreover, it mediates the repressive effect of AAs on *ilps 4, 6*, and *7* under the low AA level condition as well as the activating AA effect on *ilps 1, 2, 3, 5*, and *8* under the high-AA-level condition.

**Fig. 2.**
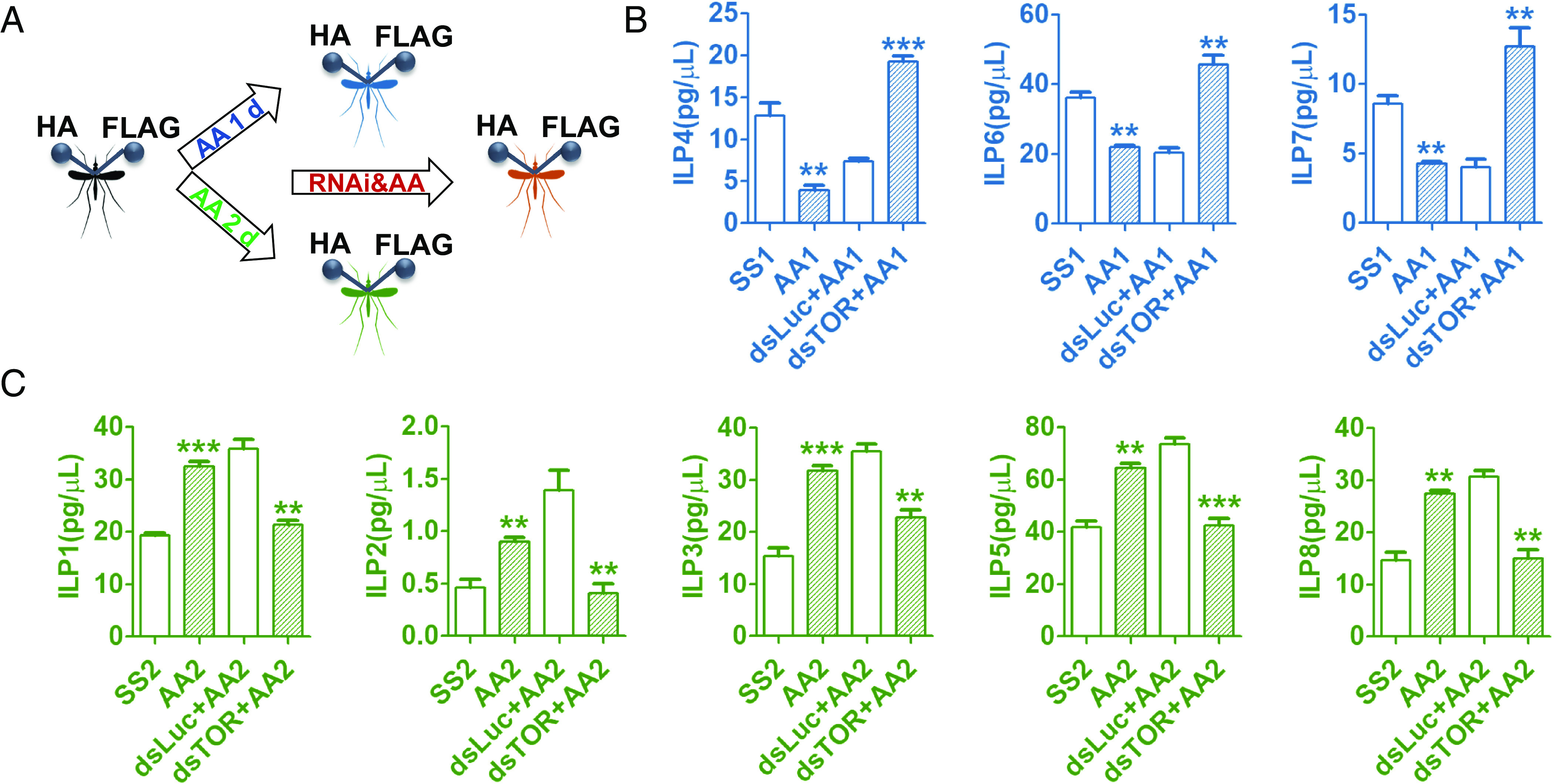
CRISPR-Cas9-mediated gene tagging for the determination of hemolymph ILP levels. (*A*) Diagram indicates AA infusion (24 h or 48 h) and TOR RNAi in epitope-tagged mosquitoes. (*B*) Hemolymph protein levels of ILPs 4, 6, and 7 were lower after the 24-h AA infusion (AA1), but higher after the 24-h AA infusion and RNAi-TOR (dsTOR+AA1), than in control SS1 (*C*) Hemolymph protein levels of ILPs 1, 2, 3, 5, and 8 were higher after the 48-h AA infusion (AA2), but lower after the 48-h AA infusion and RNAi-TOR (dsTOR+AA2), than in control SS2. Hemolymph ILP-HA/FLAG content (pg/μL) was determined using ELISA in tagged females after AA infusion or RNAi treatments and respective controls (infusion of saline solution or injection of ds*Luc*). Data represent three biological replicates with five individuals in each and are shown as mean ± SEM; *P <0.05, **P < 0.01, ***P < 0.001.

To investigate whether this pattern of the AA-TOR regulation extends to ILPs at the protein level, we generated CRISPR-Cas9 epitope-tagged ILPs in *Ae. aegypti*. As previously described, hemagglutinin (HA) and FLAG tags were fused into the B- and A- chains in each of eight ILPs using CRISPR-Cas9-mediated homology-directed repair and single-stranded oligodeoxynucleotides (34). We measured the hemolymph level of each epitope-tagged ILP by enzyme-linked immunosorbent assay (ELISA) in the CRISPR-Cas9 modified mosquito females. The hemolymph levels of ILPs 4, 6, and 7 were down-regulated after the 24-h AA infusion in the epitope-tagged 3-d-old female adults, while those of ILPs 1, 2, 3, 5, and 8 were up-regulated after the 48-h AA infusion (Fig. 2). Next, we performed AA infusion in combination with TOR RNAi. *dsTOR* was injected to the epitope-tagged females at 6 h PE and after 3 d of recovery, these mosquitoes were infused with AA (24 h or 48 h; 0.083 μL/h). We found that the hemolymph levels of ILPs 4, 6, and 7 were higher in AA- *dsTOR* RNAi mosquitoes (Fig. 2). In contrast, the hemolymph levels of ILPs 1, 2, 3, 5, and 8 were lower than those observed with *dsLuc* as a control (Fig. 2). The amounts of ILP proteins in the circulating hemolymph varied from 0.4 to 80 pg/μL.

### GATAa and GATAr Are Critical for the Control of *ilp* Gene Expression.

**Fig. 3.**
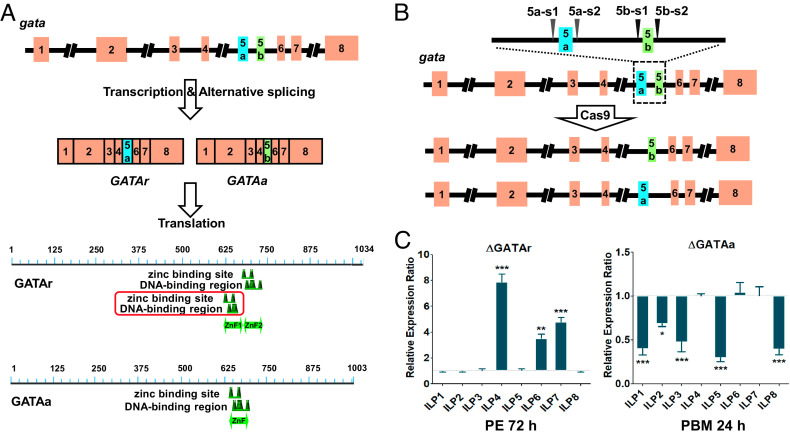
Generation of isoform-specific CRISPR-Cas9 gene editing of GATA isoforms. (*A*) Diagram indicates that *gata* gene undergoes alternative splicing of its pre-mRNA, producing two isoform-specific variants, GATAr and GATAa. GATAr factor has an extra binding region compared with GATAa. (*B*) Schematic of sgRNA (upstream 5a-s1 and downstream 5a-s2) cleavage used for deletion of the fifth exon “exon-5a” of GATAr upon sgRNA (upstream 5b-s1 and downstream 5b-s2) cleavage of GATAa “exon-5b”. (*C*) Isoform-specific knockout of GATAr (*ΔGATAr*) activated *ilp* transcripts *46*, and *7* but displayed no significant effect on the other *ilps* before blood feeding (PE 72 h); isoform-specific knockout of GATAa (*ΔGATAa*) repressed *ilp* transcripts *1235*, and *8*, but no significant effects on the other three *ilps* after a blood meal (PBM 24 h). Wild-type females were used as control. Data represent three biological replicates with 30 individuals in each and are shown as mean ± SEM; *P < 0.05, **P < 0.01, ***P < 0.001.

Nutrition signals, particularly AAs, are reportedly transduced through TOR to GATA-type TFs, which regulate the target genes (30, 33, 35). To elucidate the mechanism and physiological function of GATA regulation of *ilp* gene expression in the mosquito, we utilized the CRISPR-Cas9 genome-editing approach to precisely disrupt GATAr and GATAa genes, respectively. In *A. aegypti* mosquitoes, these two GATA isoforms have distinct fifth exons. For generating isoform-specific CRISPR-Cas9 gene editing of GATAa and GATAr, we designed a pair of single-stranded guide RNAs (sgRNAs) (5a-s1, 5a-s2) flanking the GATAr-specific fifth (5a) exon and another pair (5b-s1 and 5b-s2) flanking the GATAa-specific exon-5b by scanning DNA sequences around the fifth exon of the *gata* gene (Fig. 3 *A* and *B*). The sgRNA target sites were complementary to the spacer part of the CRISPR RNA and had a protospacer adjacent motif of NGG (the recognition site for *Streptococcus pyogenes* Cas9) (*SI Appendix*, Fig. S2). Each synthetic sgRNA (40 ng/μL) and Cas9 protein (333 ng/μL) were injected into mosquito embryos to generate genomic disruption, as previously described (18). Sanger sequencing revealed polymorphic mutations and depletion of GATAr and GATAa fifth exon sequences (*SI Appendix*, Fig. S2).

The GATAr- and GATAa- mutated mosquitoes at 72-h PE and 24-h PBM were used for RT-qPCR analysis. The transcript levels of *ilps* in either mutations or wild-type (WT) controls were measured. The levels of the AA-down-regulated *ilps* (*ilps 4*, *6,* and *7*) were elevated but the up-regulated *ilps* had no change in *GATAr*-disrupted mosquito females at 72-h PE relative to WT. Levels of the AA-up-regulated *ilps* (*ilps 1*, *2*, *3*, *5,* and *8*) were lower and the down-regulated *ilps* had no change in *GATAa*-disrupted mosquito females at 24-h PBM relative to WT (Fig. 3*C*). This experiment shows the specificity of obtained CRIPSR-CAS9 mutants for GATAa and GATAr. Moreover, it clearly indicated the differential action of GATAa and GATAr in controlling the *ilp* gene expression.

### GATAr and GATAa Affect Egg Development.

**Fig. 4.**
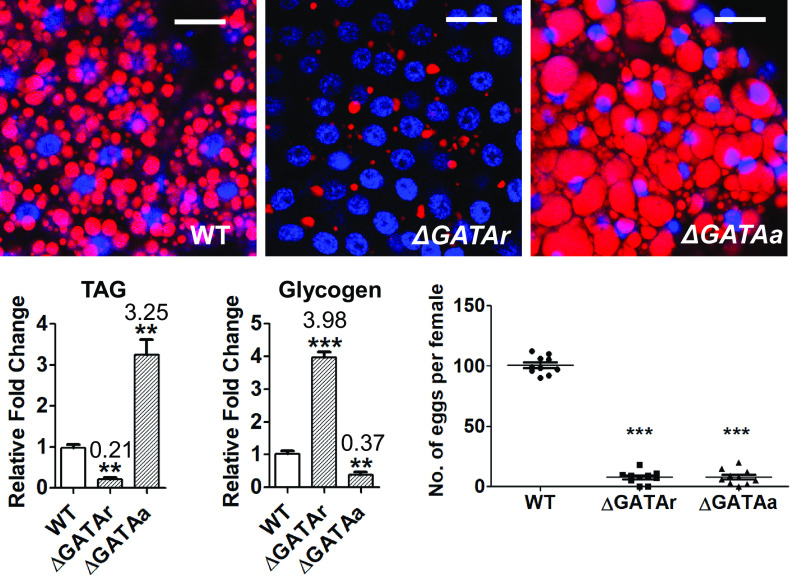
Lipid and glycogen levels, and egg numbers in WT and *gata* isoform-specific CRISPR-Cas9 mutant females. Lipid droplets in the fat body from WT and *gata* mutant females (*ΔGATAr* and *ΔGATAa*) at 24-h PBM were detected using Nile red staining and visualized under a Leica SP5 confocal microscope (scale bars, 25 μm; Blue, DAPI staining). TAG and glycogen levels and egg numbers are shown to be affected differently in mutant females than in WT controls. Data represent three biological replicates (six individuals in each replication) with three technical replicates and are shown as mean ± SEM; *P < 0.05, **P < 0.01, ***P < 0.001.

We dissected the ovaries from WT, GATAr and GATAa mutant vitellogenic mosquitoes at 24-h PBM, when the ovary had developed follicles. We found that 81% of the GATAr mutants and 72% of GATAa mutants displayed dramatic defects in ovarian development and egg maturation. Examination of the GATAr mutant ovaries revealed much smaller follicles at 24 h PBM and fewer laid eggs by the end of the mosquito gonadotrophic cycle than with WT mosquitoes (*SI Appendix*, Fig. S3 and Fig. 4). The GATAa mutant ovaries were half melanized, displayed several bigger follicles at 24-h PBM and produced fewer eggs than WT (*SI Appendix*, Fig. S3 and Fig. 4). Collectively, both GATAr and GATAa regulate *ilp* gene expression and promote egg development in female mosquitoes.

To evaluate the effect of GATAr and GATAa in regulating nutrient metabolism during mosquito reproduction, we stained the lipid droplets in the fat bodies of WT and mutants (with abnormal ovaries) at 24-h PBM with Nile red. The lipid droplets were dramatically smaller and less abundant in the GATAr than in WT control. On the contrary, they were considerably bigger and more abundant in GATAa mutant females than in WT control. (Fig. 4). Mosquito females store lipid energy reserves as TAGs in the fat body cells during the sugar-feeding (PE) phase; TAGs are required for subsequent reproductive events (36, 37). A significant difference in TAG levels in GATAr and GATAa mutants was detected compared with those in WT control mosquitoes (Fig. 4). GATAr mutant females had highly reduced TAG levels, while the GATAa mutant females displayed elevated TAG levels at 24 h PBM (Fig. 4). However, glycogen levels were greater in GATAr and lower in GATAa mutants (Fig. 4). The GATAr and GATAa mutants created by CRISPR-Cas9 emphasize crucial and differential roles of these GATA isoforms in ovarian development and in lipid and sugar homeostasis.

### GATAr Binds to *ilps 4*, *6,* and *7*, whereas GATAa Binds to *ilps 1*, *2*, *3*, *5,* and *8*.

**Fig. 5.**
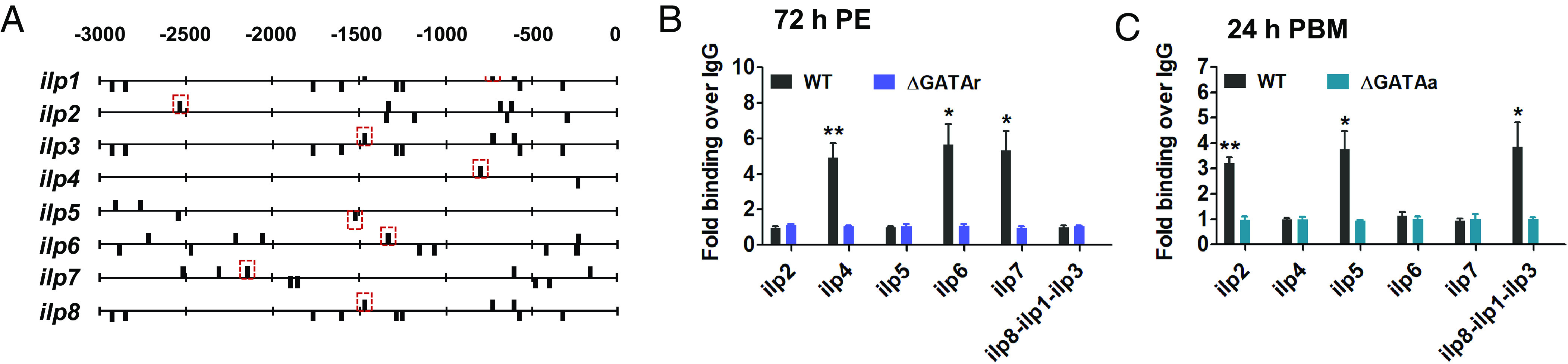
ChIP-qPCR assay revealing interaction of GATA with *ilp* genes. (*A*) Graphical representation of the *ilp* genomic loci and their GATA-motif binding sites. The dashed red boxes show the sites used for subsequent assays. (*B*) GATA genomic binding was found before blood feeding in *ilp4ilp6* and *ilp7*, but absent in the promoters of *ilp8-ilp1-ilp3* operon, *ilp2* and *ilp5*. CRISPR-Cas9 isoform-specific knockout of GATAr diminished the binding enrichment. (*C*) GATA genomic binding was found at 24 h PBM in *ilp8-ilp1-ilp3* operon, *ilp2* and *ilp5*, but absent in the promoters of *ilp4ilp6*, and *ilp7*. CRISPR-Cas9 isoform-specific knockout of GATAa diminished the binding enrichment. The relative fold enrichment of repeats was obtained from specific antibodies (anti-GATA). Data represent three biological replicates with 30 individuals in each and are shown as mean ± SEM; *P < 0.05, **P < 0.01, ***P < 0.001.

Mosquito GATAr or GATAa belong to GATA-type TFs. These are zinc-finger proteins that bind the consensus DNA sequence (A/T)GATA(A/G) in target gene regulatory regions and control the activity of these genes (30, 35). Putative GATA-binding sites are present in regulatory regions of all eight *ilp* genes of the *A. aegypti* mosquito (Fig. 5*A*). To confirm authenticity of these bindings sites, we conducted chromatin immunoprecipitation (ChIP) analysis (see *SI Appendix*, *Supplemental Section* for the method details). GATA binding was enriched at the AA-down-regulated, GATAr-repressed *ilp* genes (*ilps 4*, *6,* and *7*) in WT female mosquitoes at 72-h PE. This binding enrichment was diminished to a background level after the CRISPR-Cas9 knockout of GATAr (Fig. 5*B*). GATA binding was at the background levels in the AA-up-regulated, GATAa-activated *ilp* genes (*ilps 1*, *2*, *3*, *5*, and *8*) at 72-h PBM. In contrast, the GATA binding at this group of ilps was elevated after a blood meal (24 h PBM). The CRISPR-Cas9 disruption of GATAa diminished this binding enrichment (Fig. 5*C*). GATA binding was at the background levels in GATAr-repressed *ilp* genes (*ilps 4*, *6*, and *7*) in WT as well in the GATAa knockouts at 24 h PBM (Fig. 5*C*). These experiments revealed that GATAr and GATAa binds specifically for different *ilps*.

**Fig. 6.**
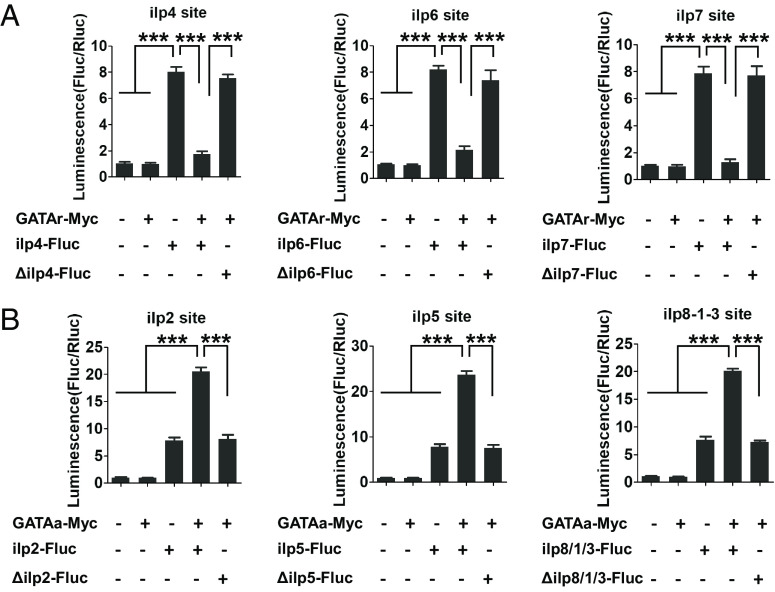
GATAr and GATAa regulate the transcription of *ilp* genes. (*A*) Luciferase reporter assay after cotransfection of expression vectors *pAc-GATAr-Myc* and reporter constructs indicates GATAr as a repressor of *ilp transcripts 46*, and *7*. (*B*) Luciferase reporter assay after cotransfection of expression vectors *pAc-GATAa-Myc* and reporter constructs indicates GATAa as a transcription activator of *ilp genes 1235*, and *8*. Treatments with no input DNA and the empty expression vector and motif mutation served as controls. Data represent six replicates and are shown as mean ± SEM; ***P < 0.001.

To gain further insight into GATAr or GATAa interactions with *ilp* genes, we performed the dual-luciferase assay in *Drosophila* Schneider 2 (S2) cells. The 1-kb 5′ upstream regulatory regions of *ilp* genes harboring the GATA-binding motif sites were subcloned into the firefly luciferase reporter vector pGL3basic (*ilp-Fluc*). They were cotransfected with either the *pAc-GATAr-Myc* or *pAc-GATAa-Myc* expression vectors into S2 cells, respectively. We detected a reduction of luciferase activity after cotransfection of *ilp4-Fluc*, *ilp6-Fluc*, or *ilp7-Fluc* with the *pAc-GATAr-Myc* expression vector. In contrast, an elevation of luciferase activity was observed after cotransfection of *ilp2-Fluc*, *ilp5-Fluc*, or *ilp8-1-3-Fluc* with the *pAc-GATAa-Myc* expression vector (Fig. 6). No significant changes were noticed after cotransfection of *ilp4-Fluc*, *ilp6-Fluc*, or *ilp7-Fluc* with the *pAc-GATAa-Myc*, or cotransfection of *ilp2-Fluc*, *ilp5-Fluc*, or *ilp8-1-3-Fluc* with *pAc-GATAr-Myc* (*SI Appendix*, Fig. S4). This experiment demonstrated binding specificities as well differential actions of GATAr and GATAa.

### Differential Effects of TOR, GATAr, and GATAa on FoxO Localization in the Fat Body Cells.

**Fig. 7.**
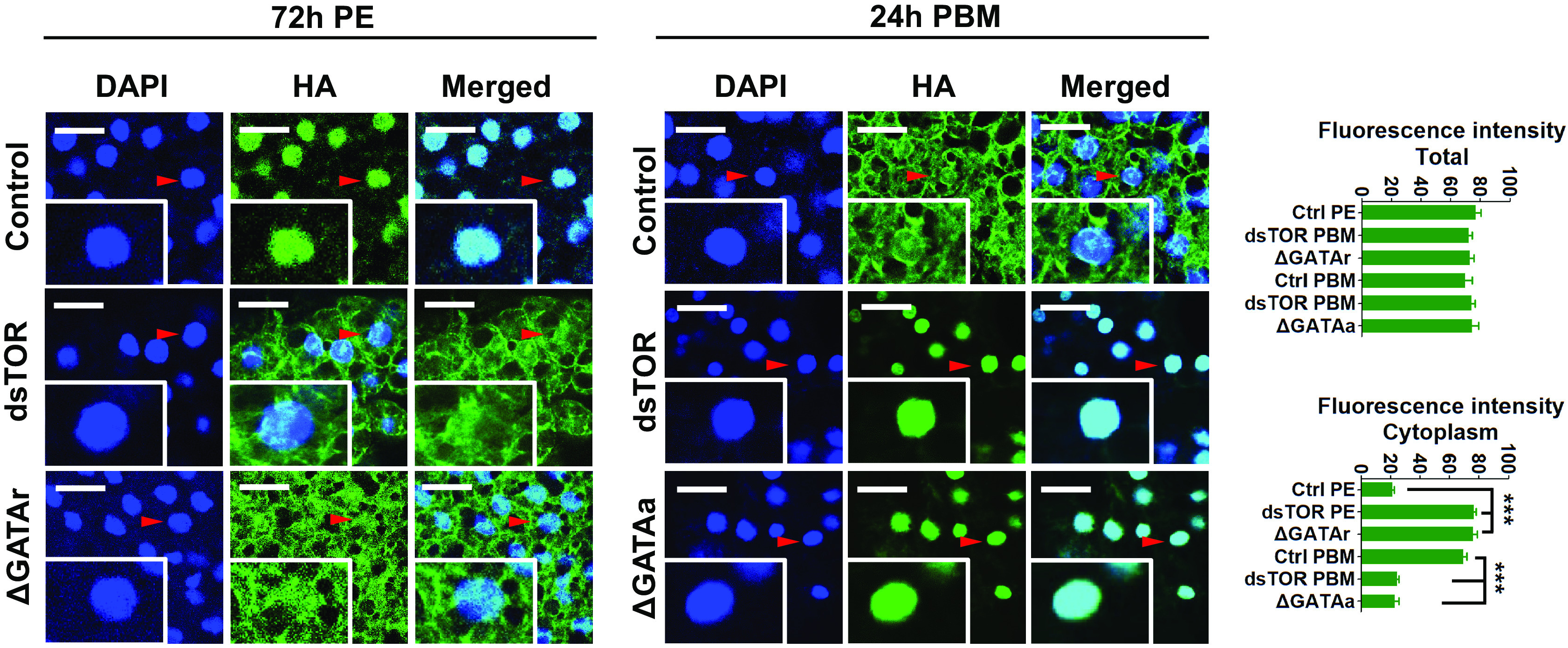
Effect of TOR and GATA isoforms on FoxO subcellular localization in fat body cells. Confocal microscopic (Leica SP5) images of the fat body cells dissected from females showing the FoxO-HA (green) and the DAPI signal (blue) (scale bar, 25 μm.) *Insets* represent images of single cells in the tissue, indicated by arrowheads. The mean fluorescence intensity of green signals indicates total and cytoplasmic FoxO-HA levels. Data represent three independent biological replicates with five images in each replicate and are shown as mean ± SEM; ***P < 0.001.

FoxO is a TF downstream of the insulin signaling pathway. Insulin binds to the insulin receptor and affects FoxO phosphorylation and cellular localization to fulfill its function (38). To study the effect of nutritional signaling on FoxO localization, we utilized the CRISPR-Cas9 homologous recombination to link the HA tag into the mosquito endogenous FoxO protein at the carboxy terminus, as previously described (18, 34). To further explore the regulation of ILP signaling and action of the TOR and GATA factors, we observed HA-tagged FoxO intracellular localization in the fat body of female mosquitoes after RNAi knockdown of TOR or CRISPR-Cas9 knockouts of GATAr or GATAa. In mosquitoes with HA-tagged FoxO in the WT background, HA-tagged FoxO is localized in fat body nuclei at 72 h PE, while predominantly in the cytoplasm at 24-h PBM (Fig. 7). The CRISPR-Cas9 mutation of GATAr in female mosquitoes with HA-tagged FoxO with HA-tagged FoxO at 72-h PE resulted in its cytoplasmic retention. In contrast, the loss of either TOR or GATAa at 24-h PBM caused FoxO-HA nuclear translocation (Fig. 7). These results indicate that the GATAr and GATAa factors have an opposite effect on FoxO cellular localization in the fat body. FoxO is activated in a GATAr-dependent manner at 72-h PE, while its action is inhibited at 24-h PBM and this is GATAa dependent.

It has been shown that FoxO binds to promoters of several carbohydrate and lipid metabolic enzyme genes in *A. aegypti*, and FoxO-RNAi elevates the transcript levels of several metabolic enzyme genes, including *succinyl-coA synthetase*, *trehalose-6-phosphate synthase*, *malate dehydrogenase*, *lipase*, and *fatty acid synthases* (34). The GATAr knockout also elevated the transcript levels of these metabolic enzyme genes, which is the opposite of the effects of the GATAa knockout or TOR-RNAi (*SI Appendix*, Fig. S5). To investigate the possibility of a direct effect of GATA on the expression of metabolic enzyme genes, we searched regulatory regions of these metabolic genes. GATA binding motifs were identified in the 5′ upstream regulatory regions of the above-mentioned metabolic enzyme genes (*SI Appendix*, Fig. S6). To determine whether GATA binds to these gene promoters, ChIP analysis was done in combination with qPCR to show GATA-bound regions. The GATA binding was undetectable at the promoters of tested metabolic enzyme genes in WT or GATA-RNAi females (*SI Appendix*, Fig. S6). This experiment revealed no direct genomic interaction between GATA factors and metabolic genes.

### FoxO Prevents GATAr Binding to *ilps 4*, *6*, and *7* Permitting Their Expression at the PE Phase.

AA-TOR dramatically activates *GATAa* gene expression after a blood meal, while the expression of the *GATAr* gene was down-regulated below a detectable level between 18-h and 24-h PBM when AAs increased to a maximal concentration in hemolymph (30, 35). To investigate the GATAa and GATAr signaling in response to TOR RNAi, we inserted HA tag at the N terminus (sequence in common except for exon-5) through CRISPR-Cas9 for intracellular imaging of mosquito GATAr and GATAa (*SI Appendix*, Fig. S7). We detected HA-tagged GATA intracellular localization in the fat body of female mosquitoes after RNAi TOR knockdown. No change in GATA signaling was observed with the loss of TOR in female mosquitoes with HA-tagged GATA at 72-h PE. In contrast, the loss of TOR at 24-h PBM caused GATA-HA reduction that correspond the regulation of TOR on GATAa while GATAr had no change (*SI Appendix*, Fig. S7).

Given the absence of any effect of TOR on GATA protein levels at 72-h PE, to determine whether FoxO is involved in the GATAr-binding of *ilp* promoters, we performed ChIP-qPCR analysis to specifically amplify GATA-bound regions before and after blood feeding in FoxO-RNAi (*dsFoxO*) or FoxO-overexpressed (*Vg-FoxO*) female mosquitoes (*SI Appendix*, Fig. S10).

We generated the binary transgenic line *Vg-Gal4/UAS-FoxO* to overexpress FoxO after a blood meal in female fat bodies according to the previously described method (39). The *UAS-FoxO* construct containing the *foxo* coding sequence was inserted into the pBac[3×P3-DsRed] plasmid at the AscI restriction site. The responder line *UAS-FoxO* was created by injecting transformation vector pBac[3×P3-DsRed, UAS-FoxO] and helper into preblastodermal embryos. The driver *Vg-Gal4* line was made previously (39). The binary transgenic mosquitoes *Vg-Gal4/UAS-FoxO* were produced by crossing the *Vg-Gal4* female (green eyes with EGFP) with the *UAS-FoxO* male (red eyes with DsRed) (*SI Appendix*, Fig. S8). FoxO overexpression during PBM in the fat body of the *Vg-Gal4/UAS-FoxO* females was detected by means of RT-qPCR and results compared with those of WT, *Vg-Gal4* and *UAS-FoxO* lines at 72-h PE, 12-h and 24-h PBM, as controls (*SI Appendix*, Fig. S8). GATA-binding was present and increased at the GATAr-repressed *ilp* genes (*ilps 4*, *6*, and *7*) after RNAi knockdown of *FoxO* before blood feeding (*SI Appendix*, Fig. S9*A*). GATA-binding was present at all *ilp* genes after blood meal at 12-h PBM, but FoxO overexpression diminished the binding enrichment at the GATAr-repressed *ilp* genes (*ilps 4*, *6*, and *7*) (*SI Appendix*, Fig. S9*B*). In contrast, no change was observed at the GATAa-activated *ilp* genes (*ilps 8-1-3*, *2*, and *5*) at 12-h PBM or 24-h PBM after FoxO overexpression when compared with the control *UAS-FoxO* female mosquitoes (*SI Appendix*, Fig. S9 *B* and C). Thus, the FoxO action is essential for preventing GATAr binding to *ilps 4, 6,* and *7* and permitting these *ilps* to be expressed at the PE phase.

**Fig. 8.**
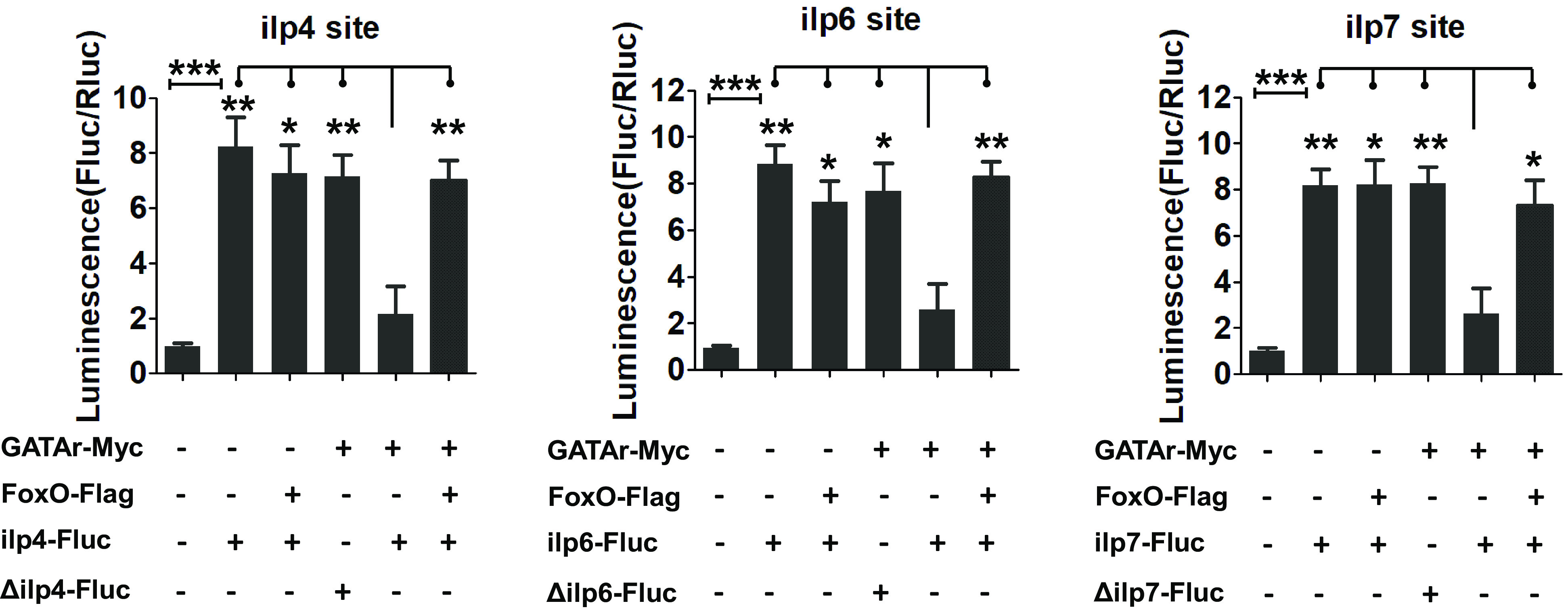
FoxO effect on the GATAr-mediated gene repression of the *ilp* promoters. Luciferase reporter assays after cotransfection of expression vectors *GATA-Myc* and/or *FoxO-Flag* along with the desired reporter constructs. Treatments with no input DNA and empty expression vector and motif mutation served as controls. Data represent six replicates and are shown as mean ± SEM; *P < 0.05, **P < 0.01, ***P < 0.001.

To gain further confirmation of the involvement of FoxO in repression of GATAr-*ilp* binding, we constructed the *pAc-FoxO-Flag* expression plasmid. The luciferase activities were recovered when S2 cells were cotransfected with the *pAc-FoxO-Flag*, *pAc-GATAr-Myc*, and the *ilp4-Fluc*, *ilp6-Fluc*, or *ilp7-Fluc* vectors and compared with the control group transfected with only *pAc-GATAr-Myc* and the *ilp4-Fluc*, *ilp6-Fluc*, or *ilp7-Fluc* vectors. This experiment demonstrated that FoxO blocked GATAr binding to these *ilps* (Fig. 8).

## Discussion

Anautogeny is a fundamental phenomenon underlying the vectorial capacity of mosquitoes (40). The gonadotrophic cycle of anautogenous female mosquitoes is separated into two phases: PE and PBM. During this cycle, the female mosquito shifts its feeding from nectar during PE to vertebrate blood in PBM to coordinate the nutrient intake and utilization with the reproductive needs for egg production (10, 11). A blood meal is digested and absorbed by mosquito females to provide nutrients as well as signaling AAs for activating the TOR pathway (29). TOR mediates AA signaling in mosquito anautogeny as its RNAi knockdown is significantly impacting mosquito egg development (41). Blood feeding activates the brain to release ovary ecdysteroidogenic hormone (OEH) and ILP3 for initiating reproductive events, and ILP3 but not OEH stimulates digestion of the blood meal (42). ILP signaling plays a critical role in regulating multiple physiological processes such as growth, metabolism, reproduction, and lifespan in animals (16, 43). Our study focused on the control of ILPs due to their importance as regulators linking metabolism and reproduction in the disease vector *A. aegypti*.

Here, we investigated the effect of the AA-TOR signaling on ILPs during the female mosquito reproductive cycle. *ilp 4*, *6*, and 7 are up-regulated during the PE phase and down-regulated after blood feeding, while *ilps 1*, *2*, *3*, *5*, and *8* exhibited high levels of expression during the PBM phase (*SI Appendix*, Fig. S1) (31). Infusion of a balanced AA mixture into PE mosquitoes has been reported to be sufficient to activate egg development (32). Our results indicate that the AA-TOR signaling pathway regulates eight *Aedes* ILPs differentially in reproducing female mosquitoes. We show that infusion of the low level of AA down-regulated the expression of *ilp 4, 6*, and *7* genes, but this AA level was not sufficient to up-regulate the expression of other ilps (1, 2, 3, 5, and 8). The activation of the latter group of the *ilp* genes required higher levels of AAs. RNAi knockdown of TOR caused the opposite effect up-regulating *ilps* 4, 6, and 7 and down-regulating *ilps* 1, 2, 3, 5, and 8. This indicated that TOR mediates the AA signaling.

Using the CRIPSR-Cas9 approach, we successfully obtained mosquito knockout mutants for the GATA isoforms GATAr and GATAa. This study has revealed that the GATA isoforms GATAr and GATAa differentially affect the expression of *ilp* genes. GATAr and GATAa factors directly interact with promoters of the *ilp* genes; GATAr specifically binds *ilps 4, 6*, and *7,* while GATAa – *ilps 1, 2, 3, 5*, and *8*. Our previous studies have shown that CRISPR-Cas9-mediated mutation on different *ilp* genes causes a differential degree of deficiency in growth, nutrient storage, carbohydrate/lipid balance and egg development (18, 19). Here, we demonstrate that *ilp4*, *ilp6*, and *ilp7* are negatively regulated by the AA-TOR signaling through GATAr factor, where GATAr inhibits the expression of these genes at the low levels of AAs. In contrast, the GATAa factor activated the expression of *ilp1*, *ilp2*, *ilp3*, *ilp5*, and *ilp8* genes by directly interacting with their promoters at the high influx of AAs during the PBM phase. Depletion of GATAr by the isoform-specific CRISPR-Cas9 resulted in the formation of small ovarian follicles and little lipid accumulation. The situation was reversed in the isoform-specific GATAa-depleted mosquitoes. Mosquito reproductive defects with enlarged ovarian follicles and excessive lipid storage were observed in the GATAa CRISPR-Cas9 mutants. Furthermore, we found that GATAr and GATAa affected the sugar reserves. Therefore, the TOR–GATA axis plays a key role in regulating adaptive physiological responses to nutrient availability by controlling ILPs in mosquitoes, and the GATA isoform–specific switch differentially mediates the AA-TOR signaling to the *ilp* genes supporting ovarian development and energy reserve homeostasis.

The secretion of brain ILPs is regulated by several TOR-dependent factors (44–46). We determined whether the levels of circulating ILPs were regulated by the AA-TOR-GATA pathway in female mosquitoes. We generated CRISPR-Cas9 epitope-tagged ILPs in *A. aegypti* female mosquitoes to analyze the abundance of each ILP protein in hemolymph. Our experiments demonstrated that indeed the levels of circulating ILPs in mosquitoes are tightly controlled by AAs, TOR, and GATA factors.

The FoxO TF activity correlates with its intracellular localization, which is controlled by the insulin signaling (47, 48). In the fat body of female adult mosquitoes, we found that FoxO was in the nuclei at 72-h PE and in the cytoplasm at 24-h PBM, when the AA level was high. This demonstrates that FoxO was active during the PE phase and inactive during the PBM one. CRISPR-Cas9 disruption of GATAr-isoform caused FoxO retardation in the cytoplasm before blood feeding at 72-h PE GATAa on FoxO. GATAa-isoform disruption resulted in FoxO nuclear localization after a blood meal at 24-h PBM. RNAi TOR knockdown caused FoxO nuclear localization like that of GATAa. Thus, GATAr and GATAa relay the AA-TOR signaling in opposite ways affecting the FoxO intracellular localization and, therefore, its activity. We showed that the knockout of GATAr isoform increases the expression of FoxO-repressed metabolic enzyme genes, while the knockout of GATAa decreases these genes. However, no direct interaction of GATAa or GATAr with these metabolic genes was detected.

**Fig. 9.**
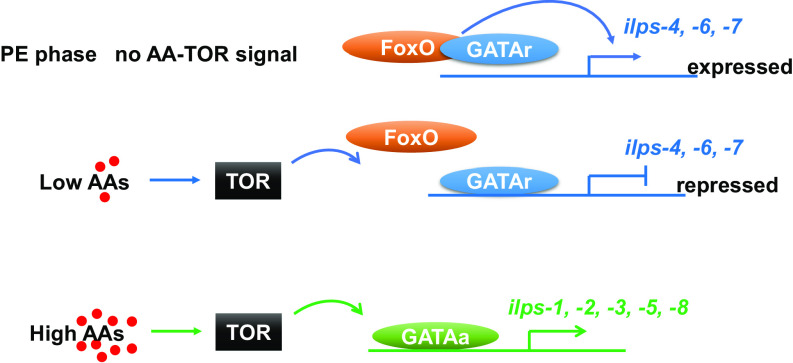
Schematic diagram of the AA-TOR-GATA axis regulating expression of *ilp* genes. FoxO is active during the PE phase, it blocks binding of GATAr to the promoters of *ilps 3, 4,* and *7*, thereby preventing their inhibition and permitting expression. The influx of AAs, even at the low dose, deactivate FoxO, and GATAr blocks the expression of these *ilp* genes. The high AA level triggers activation of *ilps 1, 2, 3, 5,* and *8.* This AA trigger is mediated by TOR and GATAa.

Our experiments further revealed the critical role of FoxO in regulating GATAr binding. We show that when FoxO is active during the PE phase, it blocks binding of GATAr to the promoters of *ilp 3, 4*, and *7*, thereby preventing their inhibition and permitting expression. The influx of AAs, even at the low dose, deactivate FoxO, and GATAr blocks the expression of these *ilp* genes. The high AA level triggers activation of *ilps 1, 2, 3, 5*, and *8.* This AA trigger is mediated by TOR and GATAa. The diagram summarizing the model of interactions of these factors during mosquito reproduction is presented in Fig. 9.

In conclusion, we have identified the TOR–GATA switch mechanism for the AA signaling control on the *ilp* gene expression and circulating ILPs, coordinating nutrient metabolism during reproductive cycles of the disease vector *A. aegypti*.

## Materials and Methods

A detailed description of the materials and methods used for this study is provided in (*SI Appendix*, Supplemental Materials and Methods**). *A. Aegypti* mosquitoes and their genetics were used. RNAi, CRISPR-Cas9 knockin, gene-tagging, isoform-specific CRISPR-Cas9 knockout, piggyBac transformation, AA infusion, ChIP, cell luciferase, ELISA, and immunofluorescence were performed. dsRNA injection, mosquito embryo microinjection, and cell culture were described previously.

## Supplementary Material

Appendix 01 (PDF)

## Data Availability

All study data are included in the article and/or *SI Appendix*.

## References

[1] B. Chala, F. Hamde, Emerging and re-emerging vector-borne infectious diseases and the challenges for control: A review. Front. Public Health **9**, 715759 (2021). 34676194 10.3389/fpubh.2021.715759 PMC8524040

[2] I. K. Moise, L. R. Ortiz-Whittingham, V. Omachonu, M. Clark, R. D. Xue, Fighting mosquito bite during a crisis: Capabilities of Florida mosquito control districts during the COVID-19 pandemic. Bmc Public Health **21**, 687 (2021). 33832475 10.1186/s12889-021-10724-w PMC8027982

[3] B. G. V. Boze, D. M. Markowski, D. Bennett, M. G. Williams, Preparations and activities necessary for aerial mosquito control after hurricanes. J. Am. Mosquito Contr. **36**, 90–97 (2020). 10.2987/19-6881.1 33647138

[4] B. Caputo, M. Manica, Mosquito surveillance and disease outbreak risk models to inform mosquito -control operations in Europe. Curr. Opin. Insect. Sci. **39**, 101–108 (2020). 32403040 10.1016/j.cois.2020.03.009

[5] A. D. T. Barrett, S. Higgs, Yellow fever: A disease that has yet to be conquered. Annu. Rev. Entomol. **52**, 209–229 (2007). 16913829 10.1146/annurev.ento.52.110405.091454

[6] O. J. Brady, S. I. Hay, The global expansion of dengue: How *Aedes aegypti* mosquitoes enabled the first pandemic arbovirus. Annu. Rev. Entomol. **65**, 191–208 (2020). 31594415 10.1146/annurev-ento-011019-024918

[7] C. J. McNeil, A. K. Shetty, Zika virus: A serious global health threat. J. Trop. Pediatrics **63**, 242–248 (2017). 10.1093/tropej/fmw080 27923889

[8] K. A. Tsetsarkin, R. B. Chen, S. C. Weaver, Interspecies transmission and chikungunya virus emergence. Curr. Opin. Virol. **16**, 143–150 (2016). 26986235 10.1016/j.coviro.2016.02.007 PMC4824623

[9] S. C. Weaver , Zika virus: History, emergence, biology, and prospects for control. Antiviral Res. **130**, 69–80 (2016). 26996139 10.1016/j.antiviral.2016.03.010 PMC4851879

[10] G. M. Attardo, I. A. Hansen, A. S. Raikhel, Nutritional regulation of vitellogenesis in mosquitoes: Implications for anautogeny. Insect Biochem. Mol. **35**, 661–675 (2005). 10.1016/j.ibmb.2005.02.013 15894184

[11] W. A. Foster, Mosquito sugar feeding and reproductive energetics. Annu. Rev. Entomol. **40**, 443–474 (1995). 7810991 10.1146/annurev.en.40.010195.002303

[12] S. Roy , Regulation of gene expression patterns in mosquito reproduction. PLoS Genet. **11**, e1005450 (2015). 26274815 10.1371/journal.pgen.1005450 PMC4537244

[13] A. R. Saltiel, C. R. Kahn, Insulin signalling and the regulation of glucose and lipid metabolism. Nature **414**, 799–806 (2001). 11742412 10.1038/414799a

[14] A. Santoro, T. E. McGraw, B. B. Kahn, Insulin action in adipocytes, adipose remodeling, and systemic effects. Cell Metab. **33**, 748–757 (2021). 33826917 10.1016/j.cmet.2021.03.019 PMC8078167

[15] D. R. Nassel, J. Vanden Broeck, Insulin/IGF signaling in *Drosophila* and other insects: Factors that regulate production, release and post-release action of the insulin-like peptides. Cell Mol. Life Sci. **73**, 271–290 (2016). 26472340 10.1007/s00018-015-2063-3 PMC11108470

[16] A. Sharma, A. B. Nuss, M. Gulia-Nuss, Insulin-like peptide signaling in mosquitoes: The road behind and the road ahead. Front. Endocrinol. **10**, 166 (2019). 10.3389/fendo.2019.00166 PMC6448002 30984106

[17] S. Chowanski , Insulin-like peptides and cross-talk with other factors in the regulation of insect metabolism. Front. Physiol. **12**, 701203 (2021). 34267679 10.3389/fphys.2021.701203 PMC8276055

[18] L. Ling, V. A. Kokoza, C. Y. Zhang, E. Aksoy, A. S. Raikhel, MicroRNA-277 targets insulin-like peptides 7 and 8 to control lipid metabolism and reproduction in *Aedes aegypti* mosquitoes. Proc. Natl. Acad. Sci. U.S.A. **114**, E8017–E8024 (2017). 28874536 10.1073/pnas.1710970114 PMC5617303

[19] L. Ling, A. S. Raikhel, Serotonin signaling regulates insulin-like peptides for growth, reproduction, and metabolism in the disease vector *Aedes aegypti*. Proc. Natl. Acad. Sci. U.S.A. **115**, E9822–E9831 (2018). 30275337 10.1073/pnas.1808243115 PMC6196551

[20] W. H. Marquardt, Biology of Disease Vectors (Academic Press, Burlington, MA, 2004).

[21] D. Y. Boudko , Substrate specificity and transport mechanism of amino-acid transceptor Slimfast from *Aedes aegypti*. Nat. Commun. **6**, 8546 (2015). 26449545 10.1038/ncomms9546 PMC4608377

[22] V. K. Carpenter , SLC7 amino acid transporters of the yellow fever mosquito *Aedes aegypti* and their role in fat body TOR signaling and reproduction. J. Insect. Physiol. **58**, 513–522 (2012). 22266018 10.1016/j.jinsphys.2012.01.005 PMC3322257

[23] E. L. Arrese, J. L. Soulages, Insect fat body: Energy, metabolism, and regulation. Annu. Rev. Entomol. **55**, 207–225 (2010). 19725772 10.1146/annurev-ento-112408-085356 PMC3075550

[24] C. Sim, D. L. Denlinger, Insulin signaling and the regulation of insect diapause. Front. Physiol. **4**, 189 (2013). 23885240 10.3389/fphys.2013.00189 PMC3717507

[25] G. Y. Wu, Functional amino acids in nutrition and health. Amino Acids **45**, 407–411 (2013). 23595206 10.1007/s00726-013-1500-6

[26] M. Galikova, P. Klepsatel, Obesity and aging in the *Drosophila* model. Int. J. Mol. Sci. **19**, 1896 (2018). 29954158 10.3390/ijms19071896 PMC6073435

[27] N. Okamoto, N. Yamanaka, Nutrition-dependent control of insect development by insulin-like peptides. Curr. Opin. Insect Sci. **11**, 21–30 (2015). 26664828 10.1016/j.cois.2015.08.001 PMC4671074

[28] M. J. Texada, T. Koyama, K. Rewitz, Regulation of body size and growth control. Genetics **216**, 269–313 (2020). 33023929 10.1534/genetics.120.303095 PMC7536854

[29] S. G. Roy, A. S. Raikhel, The small GTPase Rheb is a key component linking amino acid signaling and TOR in the nutritional pathway that controls mosquito egg development. Insect Biochem. Mol. **41**, 62–69 (2011). 10.1016/j.ibmb.2010.10.001 PMC3022117 21035549

[30] J. H. Park, G. M. Attardo, I. A. Hansen, A. S. Raikhel, GATA factor translation is the final downstream step in the amino acid/target-of-rapamycin-mediated vitellogenin gene expression in the anautogenous mosquito *Aedes aegypti*. J. Biol. Chem. **281**, 11167–11176 (2006). 16490782 10.1074/jbc.M601517200

[31] J. Colombani , A nutrient sensor mechanism controls *Drosophila* growth. Cell **114**, 739–749 (2003). 14505573 10.1016/s0092-8674(03)00713-x

[32] K. Uchida , Induction of oogenesis in mosquitoes (*Diptera: Culicidae*) by infusion of the hemocoel with amino acids. J. Med. Entomol. **38**, 572–575 (2001). 11476338 10.1603/0022-2585-38.4.572

[33] G. M. Attardo, S. Higgs, K. A. Klingler, D. L. Vanlandingham, A. S. Raikhel, RNA interference-mediated knockdown of a GATA factor reveals a link to anautogeny in the mosquito *Aedes aegypti*. Proc. Natl. Acad. Sci. U.S.A. **100**, 13374–13379 (2003). 14595016 10.1073/pnas.2235649100 PMC263821

[34] L. Ling, A. S. Raikhel, Cross-talk of insulin-like peptides, juvenile hormone, and 20-hydroxyecdysone in regulation of metabolism in the mosquito *Aedes aegypti*. Proc. Natl. Acad. Sci. U.S.A. **118**, e2023470118 (2021). 33526700 10.1073/pnas.2023470118 PMC8017721

[35] D. Martin, M. D. Piulachs, A. S. Raikhel, A novel GATA factor transcriptionally represses yolk protein precursor genes in the mosquito *Aedes aegypti* via interaction with the CtBP corepressor. Mol. Cell Biol. **21**, 164–174 (2001). 11113191 10.1128/MCB.21.1.164-174.2001 PMC88790

[36] H. Briegel, A. Hefti, E. DiMarco, Lipid metabolism during sequential gonotrophic cycles in large and small female *Aedes aegypti*. J. Insect Physiol. **48**, 547–554 (2002). 12770082 10.1016/s0022-1910(02)00072-0

[37] R. Ziegler, M. M. Ibrahim, Formation of lipid reserves in fat body and eggs of the yellow fever mosquito *Aedes aegypti*. J. Insect Physiol. **47**, 623–627 (2001). 11249951 10.1016/s0022-1910(00)00158-x

[38] D. Accili, K. C. Arden, FoxOs at the crossroads of cellular metabolism, differentiation, and transformation. Cell **117**, 421–426 (2004). 15137936 10.1016/s0092-8674(04)00452-0

[39] V. A. Kokoza, A. S. Raikhel, Targeted gene expression in the transgenic *Aedes aegypti* using the binary Gal4-UAS system. Insect Biochem. Mol. **41**, 637–644 (2011). 10.1016/j.ibmb.2011.04.004 PMC3124619 21536128

[40] A. S. Raikhel , Molecular biology of mosquito vitellogenesis: From basic studies to genetic engineering of antipathogen immunity. Insect Biochem. Mol. **32**, 1275–1286 (2002). 10.1016/s0965-1748(02)00090-5 12225918

[41] I. A. Hansen, G. M. Attardo, J. H. Park, Q. Peng, A. S. Raikhel, Target of rapamycin-mediated amino acid signaling in mosquito anautogeny. Proc. Natl. Acad. Sci. U.S.A. **101**, 10626–10631 (2004). 15229322 10.1073/pnas.0403460101 PMC489984

[42] K. J. Vogel, M. R. Brown, M. R. Strand, Ovary ecdysteroidogenic hormone requires a receptor tyrosine kinase to activate egg formation in the mosquito *Aedes aegypti*. Proc. Natl. Acad. Sci. U.S.A. **112**, 5057–5062 (2015). 25848040 10.1073/pnas.1501814112 PMC4413300

[43] L. Boulan, M. Milan, P. Leopold, The systemic control of growth. Cold Spring Harb. Perspect. Biol. **7**, a019117 (2015). 26261282 10.1101/cshperspect.a019117 PMC4665074

[44] C. Geminard, E. J. Rulifson, P. Leopold, Remote control of insulin secretion by fat cells in *Drosophila*. Cell Metab. **10**, 199–207 (2009). 19723496 10.1016/j.cmet.2009.08.002

[45] N. Agrawal , The *Drosophila* TNF Eiger is an adipokine that acts on insulin-producing cells to mediate nutrient response. Cell Metab. **23**, 675–684 (2016). 27076079 10.1016/j.cmet.2016.03.003

[46] R. Delanoue , *Drosophila* insulin release is triggered by adipose Stunted ligand to brain Methuselah receptor. Science **353**, 1553–1556 (2016). 27708106 10.1126/science.aaf8430

[47] S. Ogg , The Fork head transcription factor DAF-16 transduces insulin-like metabolic and longevity signals in *C. elegans*. Nature **389**, 994–999 (1997). 9353126 10.1038/40194

[48] O. Puig, M. T. Marr, M. L. Ruhf, R. Tjian, Control of cell number by *Drosophila* FOXO: Downstream and feedback regulation of the insulin receptor pathway. Gene Dev. **17**, 2006–2020 (2003). 12893776 10.1101/gad.1098703 PMC196255

