# Supplementary material for: Amino acid–dependent regulation of insulin-like peptide signaling is mediated by TOR and GATA factors in the disease vector mosquito Aedes aegypti
Source: Proc Natl Acad Sci U S A. 2023 Aug 14;120(34):e2303234120. doi: 10.1073/pnas.2303234120 (PMC10450652; doi:10.1073/pnas.2303234120)
Supplement: Supplementary file 1 — Appendix 01 (PDF) [file pnas.2303234120.sapp.pdf]

Supplementary Information

Materials and Methods

Experimental Animals. *Aedes aegypti* mosquitoes were maintained in insectary at 27°C and 80% humidity. Larvae were reared in water with complete larval diet (a mixture of rat chow, yeast and lactalbumin, 1:1:1 ratio), and adult mosquitoes were reared in cages with access to water and 10% (wt/vol) sucrose solution. Female mosquito adults were blood fed on White Leghorn chickens. The treatment of vertebrate animals was approved by the University of California Riverside Institutional Animal Care and Use Committee.

Amino Acids Infusion. Glass micropipettes were pulled on a puller (model P-2000, Sutter Instrument Co., U.S.A.) and mounted on the tip of the stainless needles of micro syringes. Ten micro syringes filled with the solution (17 amino acids mixture) were set on an infusion pump (model 22 MI/W, Harvard Apparatus, U.S.A.). 3-d-old female *Ae. aegypti* adults were immobilized between a sponge cube and a fine mesh cover. The sponge holding the female was fixed on a micromanipulator, and ten females were infused at the same time according to the infusion methods (33). The amino acids mixture was infused into females at the flow rates for 24 h (0.083 µl/h × 24 h = 2.0 µl/24 h) or 48 h (4.0 µl/48 h). *Aedes* saline was infused as a control.

dsRNA Synthesis and RNAi injection. For RNAi experiments, the MEGAscript Kit (Ambion) was used to synthesize dsRNA. T7 promoter sequences were added to DNA using PCR to generate templates. PCR primers are listed in Table S1. At 6 h PE, 0.3µL of 4 µg/µL dsRNA was injected into the thorax of cold-anesthetized female mosquitoes using Picospritzer II (General Valve). After 3 days recover, the injected mosquitoes were fed with blood.

RNA Extraction and qRT-PCR. Total RNA was extracted from mosquito tissues by using TRIzol Reagent (Invitrogen) method. cDNA was synthesized from 2 µg purified RNA using the SuperScript IV First-Strand Synthesis System (Invitrogen) following manufacturer's protocol. qRT-PCR was conducted using the SYBR Green Supermix (Bio-Rad) following manufacturer's protocol. Each sample was measured in triplicate. The relative expression was calculated as $2^{-\Delta\Delta Ct}$. The housekeeping gene *RPS7* was used as reference for normalization.

Embryonic Injection for CRISPR-Cas9. sgRNAs ($N_{21}GG$) were synthesized using the MEGAscript T7 Transcription Kit (Ambion) and purified using the MEGA-clear Transcription Clean-Up Kit (Ambion) following the manufacturer's instructions. Cas9 protein with nuclear localization signal (PNA Bio) was diluted to 1 mg/mL and stored at -80°C. Preblastoderm-stage embryos were lined on filter paper, desiccated slightly, and transferred onto glass slides. To generate genome disruption, a mixture of sgRNAs (40 ng/µL) and Cas9 protein (333 ng/µL) were injected into embryos in the posterior pole at an angle of 10 to 25°. The injected embryos hatched on the 5th day after injection.

CRISPR-Cas9-Mediated Epitope Tagging. CRISPR-Cas9-mediated HDR was used to generate epitope tagging. ssODN donors of 199 bases were synthesized as Ultramer DNA oligos (Integrated DNA Technologies) containing homologous arms of about 86 bases and 27-base HA-tag or 24-base FLAG-tag sequence. A mixture of ssODNs (125 ng/μL), sgRNAs (40 ng/μL), and Cas9 protein (333 ng/μL) were injected into the embryos. The injected embryos were hatched on the 5th day after injection and reared to adulthood. The female adults were crossed with wild-type males for oviposition before PCR verification. Offspring embryos from positive females were injected for next tagging, then hatched and raised to adults for genomic PCR, RNAi, AAs infusion, and ELISA analysis.

Measurements of ILP Levels Using ELISA. 20 μL of pooled hemolymph in phosphate buffered saline (PBS) was collected on ice from each female mosquito using Picospritzer II (General Valve). Five females per sample were used for ELISA. Immunoplates were coated (overnight, 4°C) with anti-FLAG antibody (Sigma-Aldrich) diluted in a buffer (0.2M sodium carbonate/bicarbonate, pH 9.4) to 5 μg/mL. The plates were washed twice with PBS-T (PBS with 0.2% Tween 20) and blocked with 2% bovine serum albumin (BSA) in PBS for 3 h at room temperature. After a triple wash with PBS-T, 5 μL of a sample or FLAG(GS)HA peptide standard (LifeTein LLC) was added to each well. 50 μL anti-HA-Peroxidase 3F10 antibody (Sigma-Aldrich, 15 ng/mL, diluted to in PBS with 1% Triton X-100) was added to each well. Plates were sealed and incubated in a humidity chamber (overnight, 4°C). The wells were washed six times with PBS-T. 1-Step Ultra TMB ELISA Substrate (Thermo Fisher Scientific) was added to each well and incubated for 20 minutes (room temperature). The reactions were stopped by adding 2M sulfuric acid. Absorbance (450 nm) was measured using a plate reader (Wallac). We used the molecular weights of 10.12 (ILP1-HA/FLAG), 9.55 (ILP2-HA/FLAG), 8.26 (ILP3-HA/FLAG), 8.17 (ILP4-HA/FLAG), 13.16 (ILP5-HA/FLAG), 17.92 (ILP6-HA/FLAG), 10.23 (ILP7-HA/FLAG) and 7.82 (ILP8-HA/FLAG) kilodaltons (kDa) for each mature protein. Three biological replicates were determined for each group.

Generating Transgenic Overexpression Mosquitoes. PBM and fat-body specific overexpression was generated by the transgenic mosquitoes *Vg-Gal4/UAS-FoxO*. The driver *Vg-Gal4* line was generated previously (39). The responder line *UAS-FoxO* was produced by injecting transformation vector pBac[3xP3-DsRed, UAS-FoxO] and helper into preblastoderm embryos from the posterior pole. The *UAS-FoxO* fragments containing the *foxo* coding sequence were inserted into the pBac[3xP3-DsRed] plasmid at the AscI restriction site. Germline transformation was conducted according to described protocols (39).

Lipid Droplet Staining. Abdominal walls with adhered fat bodies were dissected from female mosquito adults and cut along the pleural membrane. Abdominal walls with fat bodies were washed in a drop of PBS and placed in 96-well culture plates in 100 μL of 4% (vol/vol) paraformaldehyde and rinsed with PBST [0.3% (vol/vol) Triton X-100 in PBS]. These preparations were incubated in Nile red solution (20% glycerol

in PBS, with a 1:10,000 dilution of 10% Nile red in DMSO) and examined with confocal microscope (Leica SP5).

Immunofluorescence. Tissues were fixed in 100 µL of 4% (vol/vol) paraformaldehyde, washed twice with PBST [0.3% (vol/vol) Triton X-100 in PBS], kept in 3% (wt/vol) BSA-PBS-T for 1 h, incubated overnight with primary antibody mouse anti-HA (1:1,000; Abcam) at 4°C, incubated 2 h with Alex Fluro Plus 488-conjugated secondary antibody (goat anti-mouse, Invitrogen), stained by ProLong Diamond Antifade Mountain with DAPI (Invitrogen), imaged using Leica SP5 confocal laser scanning microscope, and the mean fluorescence intensity was analyzed using *ImageJ* software.

Determination of TAG Levels. TAG levels were measured using the Triglyceride Colorimetric Assay Kit (Cayman Chemical) following the manufacturer's instructions. Six mosquito females were homogenized in 100 µL of Diluent Assay Reagent. After centrifugation, 10 µL of the supernatant was transferred to the 96-well plate and incubated with Enzyme Mixture solution for 30 min at 37°C. The absorbance values were measured (Wallac Victor2), and results were calculated using the equation obtained from the standard curve.

Determination of Glycogen Levels. Glycogen assays were performed using Glycogen Assay Kit (Cayman Chemical) following the manufacturer's instructions. Six fat bodies were homogenized in 100 µL of Diluent Assay Buffer. After centrifugation, 10 µL of supernatant was incubated with Enzyme Solution for 30 min at 37°C then with Developer for 15 min at 37°C. The fluorescence values were measured (Wallac Victor2), and results were calculated using the equation obtained from the standard curve.

ChIP Analysis. ChIP assays were performed using Magna ChIP G Tissue Kit (Millipore) according to the manufacturer's instructions. Briefly, mosquito tissues were sheared in ice-cold Tissue Stabilizing Solution with Protease Inhibitor (PI), fixed using formaldehyde (1%), treated with Lysis Buffer/ PI, and sonicated on wet ice. Immunoprecipitating antibody and Protein G magnetic beads with the magnetic separator were used in immunoprecipitation of crosslinked protein/DNA. The protein/DNA crosslinks were reversed, and the DNA was purified for downstream detection (qPCR). IgG of the same species was used as a negative control. RNAi- or CRISPR-Cas9-mediated disruption was performed as a control. Experiments were done with triplicate samples with 30 females in each.

Luciferase Reporter Assay. The promoter regions were subcloned to the firefly luciferase reporter pGL3-Basic plasmid (Promega). The putative binding sites were mutated by incorporating restriction sites and In-Fusion HD Cloning (TaKaRa) in reporter plasmids. The expression plasmids were constructed by inserting the coding sequence of interest into the pAc5.1 vector with Myc or FLAG tag. The desired reporter plasmid (200 ng) and the control Renilla luciferase reporter plasmid (50 ng), with or without expression plasmid (200 ng), were co-transfected into *Drosophila* S2 cells using FuGENE HD Transfection Reagent (Promega).

Luciferase were determined at 48 h post-transfection using the Dual Luciferase Reporter Assay System (Promega) according to the manufacturer's instructions.

Statistical Analysis. All values are presented as mean ± SEM. Mean values were compared using the Student's t-test at the following significance levels: *P < 0.05, **P < 0.01, and ***P < 0.001. Statistical analyses were performed using GraphPad Prism 6.

Supplemental Figure Legends

Fig. S1. Temporal distribution of *ilp* transcripts in female *Ae. aegypti* mosquitoes (31). Relative expression profiles of *ilps 1*, *3*, *4*, *7* and *8* in heads, *ilp2* in ovaries, *ilp5* in carcasses and *ilp6* in fat bodies at 12 and 48 h PE, and at 12, 24 and 48 h PBM. Data represent three biological replicates, with 30 mosquitoes in head replication and 10 mosquitoes in other tissue replication and are shown as mean ± SEM. *P < 0.05, **P < 0.01, and ***P < 0.001.

Fig. S2. Amino acid infusion and RNAi efficiency of *TOR*. (*A*) Composition of the infused solution. (*B*) Extent of ovarian development induced by infusion of 17 amino acids into the hemocoel of female mosquitoes for 24 h (0.083 μl/h × 24 h = 2.0 μl/24 h) or 48 h (4.0 μl/48 h). (*C*) Diagram indicates AA-infusion and RNAi. qRT-PCR determination of the RNAi efficiency of *TOR*. Relative mRNA abundances of *TOR* were measured in *dsTOR*- and *dsLuc*-injected (0.3μL of 4 μg/μL) mosquitoes respectively post AAs infusion. Data represent three biological replicates and are shown as mean ± SEM ***P* < 0.01.

Fig. S3. GATAr/a isoform-specific exon 5 knockout screening. (*A*) Schematic of primers used to screen for deletion of the fifth exon of GATAr (exon-5a) upon sgRNAs cleavage upstream (5a-s1) and downstream (5a-s2). Sanger genomic sequencing of the exon-5a region for parental (blue) and exon knockout (green) are shown. (*B*) Comparison of the parental (blue) and GATAa isoform-specific (exon-5b) knockout (green) genomic sequencing around the sgRNA (5b-s1 and 5b-s2). The blue letters in the *gata* sequence represent the sgRNA target site. The red letters indicate the PAM sequence.

Fig. S4. Genomic disruption of *gata* by isoform-specific CRISPR-Cas9 results in abnormal phenotypes of ovarian development in mosquitoes. Ovaries were dissected from WT, *ΔGATAr* and *ΔGATAa* female mosquitoes at 24 h PBM. (Scale bar: 1 mm.) The dashed circles show the follicle shape. The right graph shows the average follicle size. Data represent three biological replicates with ten individuals in each and are shown as mean ± SEM. **P* <0.05, ***P* < 0.01, ****P* < 0.001.

Fig. S5. GATAa and GATAr and other *ilp* genes in cell luciferase reporter assay. (*A*) Luciferase reporter assay after co-transfection of expression vectors *pAc-GATAa-Myc* and reporter constructs indicates that GATAa has no change on *ilp4*, *ilp6* and *ilp7* promoters. (*B*) Luciferase reporter assay after co-transfection of expression vectors *pAc-GATAr-Myc* and reporter constructs indicates that GATAr has no change on *ilp8-ilp1-ilp3* operon, *ilp2* and *ilp5* promoters. Treatments with no input DNA and the empty expression vector and motif mutation served as controls. Data represent six replicates and are shown as mean ± SEM ***$P < 0.001$.

Fig. S6. Comparative analysis of transcript abundance of metabolic enzyme genes in isoform-specific knockout of *ΔGATAr* and *ΔGATAa* and *dsTOR* RNAi female mosquitoes. Isoform-specific knockout of *ΔGATAr* elevated transcripts of the metabolic enzyme genes: *succinyl-coA synthetase* (*SCS*), *trehalose-6-phosphate synthase* (*TPS*), *malate dehydrogenase* (*MDH*), *lipase*, and *fatty acid synthases* (*FAS*), similar to RNAi-*FoxO* (31). Isoform-specific knockout of *ΔGATAa* and RNAi knockdown of *TOR* repressed them, opposite to RNAi-*FoxO* (31). WT and *dsLuc* were used as controls. Data represent three biological replicates and are shown as mean ± SEM *$P < 0.05$, **$P < 0.01$, ***$P < 0.001$.

Fig. S7. The absence of GATA binding to the promoters of metabolic enzyme genes. (*A*) Graphical representation of the genomic loci of metabolic enzyme genes and their GATA-motif binding sites. (*B*) ChIP analysis of 3-kb regulatory regions shows no enrichment from anti-GATA antibodies. Data represent three biological replicates with 30 individuals in each and are shown as mean ± SEM.

Fig. S8. In vivo labeling of endogenous GATA protein by HDR. Graphical representation of the mosquito genomic loci of *gata* showing the target site for Cas9, sgRNA, and ssODN. The sequence of the sgRNA target site is labeled in red. The PAM sequence is labeled in blue. The initiation codon of *gata* is underscored. The Cas9 cleavage site is indicated by a black arrowhead. Confocal microscopic (Leica SP5) images of the fat body cells dissected from females showing the GATA-HA (red) and the DAPI signal (blue) (Scale bar: 25 μm.)

Fig. S9. Generation of the binary transgenic mosquitoes. Schematic diagrams of the pBac[3xP3-DsRed, UAS-FoxO] and pBac[3xP3-EGFP, Vg-Gal4] transgenic vectors. The *UAS-FoxO* fragments containing the *foxo* coding sequence were inserted into the pBac[3xP3-DsRed] plasmid. The responder line *UAS-FoxO* was produced by injecting pBac[3xP3-DsRed, UAS-FoxO] and helper into preblastoderm embryos. The binary transgenic mosquitoes *Vg-Gal4/UAS-FoxO* were

produced by crossing the *Vg-Gal4* female with the *UAS-FoxO* male. Fluorescence images of *Vg-Gal4/UAS-FoxO* mosquitoes show green eyes under EGFP microscope filter and red eyes under DsRed filter and the white light as a control. FoxO overexpression during PBM in the fat body of the *Vg-Gal4/UAS-FoxO* females (*Vg-FoxO*) were detected by RT-qPCR, compared with WT, *Vg-Gal4* and *UAS-FoxO* lines at 12 h or 24 h PBM and compared with them at 72 h PE as controls.

Fig. S10. FoxO is required for the GATAr-bound *ilps* as a role of interrupter. (*A*) ChIP-qPCR assay for GATA and *ilp* genes in WT and *dsFoxO*-RNAi females before blood feeding at 72 h PE when the binding enrichment was found only in *ilp4*, *ilp6* and *ilp7* (as we mentioned in Fig. 5). RNAi-*FoxO* increased the GATA-binding enrichment in the promoters of *ilp4*, *ilp6* and *ilp7* (GATAr-*ilps*). (*B*) ChIP-qPCR assay for GATA and *ilp* genes in *UAS-FoxO* and *Vg-FoxO* females after a blood meal (12 h PBM). Transgenic FoxO overexpression (*Vg-FoxO*) diminished the binding enrichment in *ilp4*, *ilp6* and *ilp7* (GATAr-*ilps*) promoters, whereas FoxO overexpression has no change on *ilp8-ilp1-ilp3* operon, *ilp2* and *ilp5* (GATAa-*ilps*) promoters after a blood meal at 12 h PBM or (*C*) 24 h PBM. The relative fold enrichment of repeats was obtained from specific antibodies (anti-GATA). Data represent three biological replicates with 30 individuals in each and are shown as mean ± SEM **P* < 0.05, ***P* < 0.01, ****P* < 0.001.

Table S1. Primers used for synthesis of sgRNA, dsRNA templates and ssODN sequences (5'-3').

Primer name	*Sequence (5'-3')*
CRISPR-S1-Forward-5a	GAAATTAATACGACTCACTATAGGTCATATACTATGTAATTAAAGTTTTAGAGCTAGAAATAGC
CRISPR-S2-Forward-5a	GAAATTAATACGACTCACTATAGGCTCATTTCGTTTTGCTAAATGTTTTAGAGCTAGAAATAGC
CRISPR-S1-Forward-5b	GAAATTAATACGACTCACTATAGGAAACGTCTAAAGCATCTAGTTTTAGAGCTAGAAATAGC
CRISPR-S2-Forward-5b	GAAATTAATACGACTCACTATAGGTCAGTCAACCTACCAGTCTTGTTTTAGAGCTAGAAATAGC
CRISPR-Forward-gata	GAAATTAATACGACTCACTATAGGTAGTTCTTACCCGCCATGTTGTTTTAGAGCTAGAAATAGC
ssODN	TGTGCGCGCCTTGGTGACATACTGTGTGAATCTTGATAACCTGACTGTGACGTGGAAGCGTAAATTTGCTGAAAAGTGTTCCAAACTACCCATACGATGTTCCAGATTACGCTATGGCGGGTAAGAACTATTTTACGAGTTTTGTTTAATAAGCGTTTAGAGATCCCAGCTACACATGTTGTGCCGGCGGGGCATGACA
CRISPR Universal reverse primer	AAAAGCACCGACTCGGTGCCACTTTTTCAAGTTGATAAC

	GGACTAGCCTTATTTTAACTTGCTATTTCTAGCTCTAAAAC
F1	GCGCTTATGCGTGAAGTAGAAC
R1	CGTCTAAAGCATCTACGGAACA
F2	ATTGGAAATTGCTTGCTTTGAGA
R2	TGCACATTAATATGAGAGCTTG
RNAi-TOR-F	TAATACGACTCACTATAGGGAGATTCGCAGAGAGAGGGAACCAAGC
RNAi-TOR-R	TAATACGACTCACTATAGGGAGACGACTGAATCACGTAGGGTGA

FIG. S1

A

Amino acids	g/100 ml solution (10%, wt:vol)
Alanine	0.77
Arginine	0.45
Asparagine	1.03
Cysteine	0.21
Glutamic acid	1.26
Glycine	0.38
Histidine	0.66
Isoleucine	0.34
Leucine	0.69
Lysine	1.00
Methionine	0.14
Phenylalanine	0.74
Proline	0.51
Serine	0.56
Threonine	0.37
Tryptophan	0.20
Valine	0.69

B

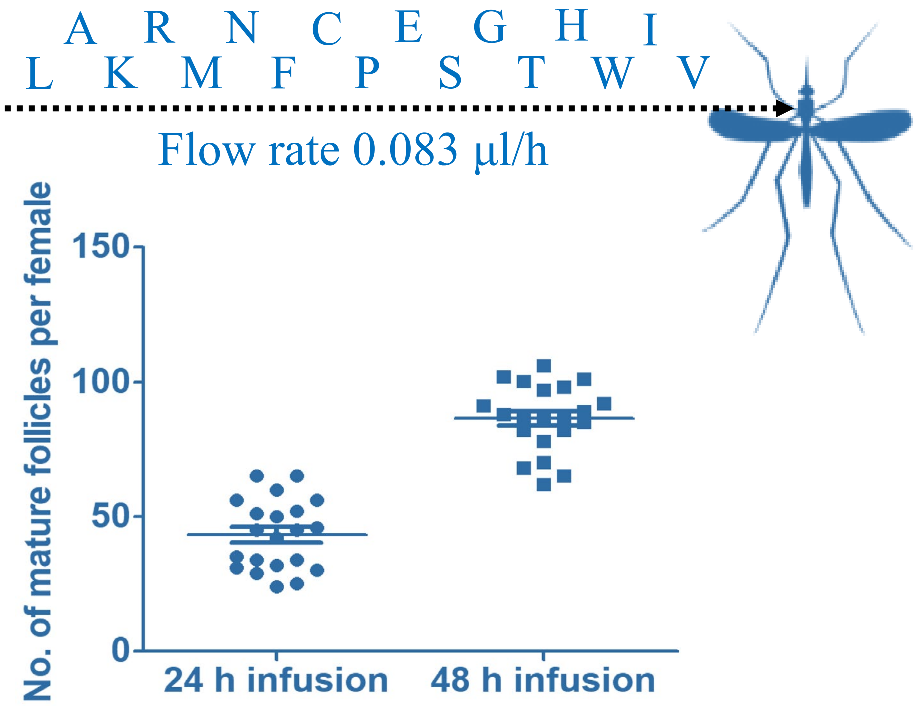

C

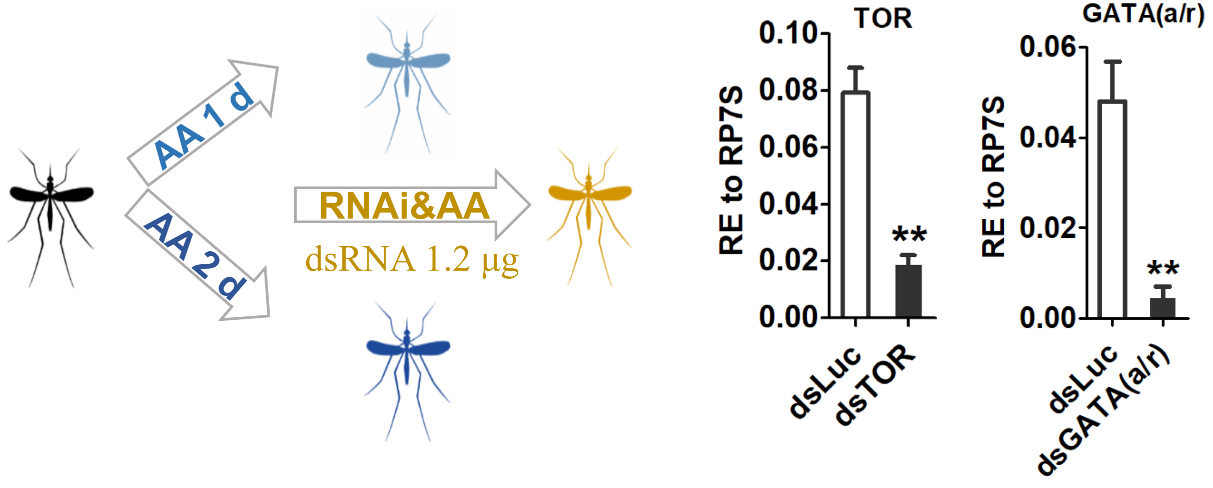

FIG. S2

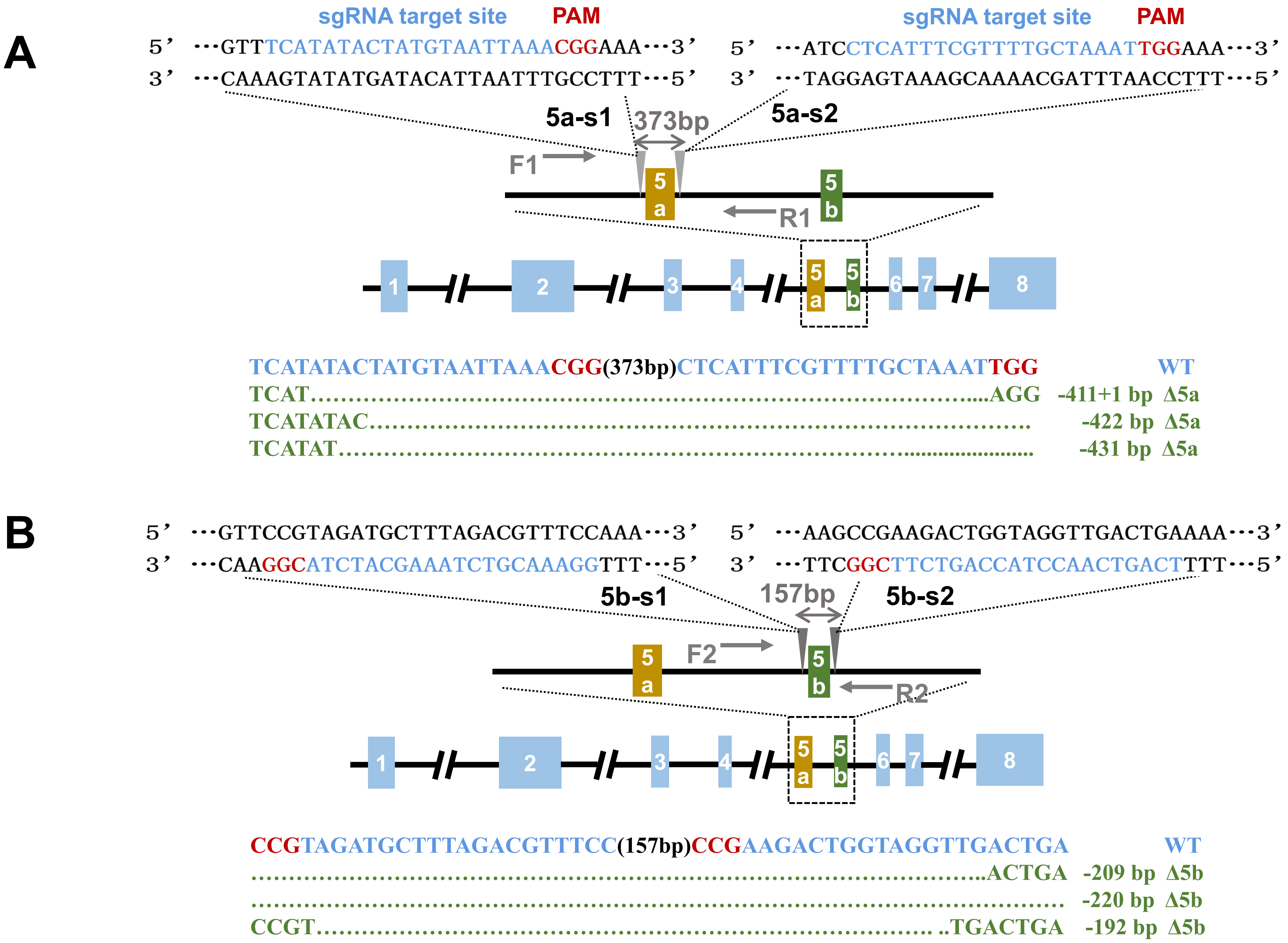

A
sgRNA target site
PAM
5’ ···GTTTCATATACTATGTAATTAAACGGAAA···3’
3’ ···CAAAGTATATGATACATTAATTTGCCTTT···5’
sgRNA target site
PAM
5’ ···ATCCTCATTTCGTTTTGCTAAATTGGAAA···3’
3’ ···TAGGAGTAAAGCAAAACGATTTAACCTTT···5’
5a-s1
373bp
5a-s2
F1
R1
5 a
5 b
1
2
3
4
6
7
8
TCATATACTATGTAATTAAACGG(373bp)CTCATTTCGTTTTGCTAAATTGG WT
TCAT.....AGG -411+1 bp Δ5a
TCATATAC..... -422 bp Δ5a
TCATAT..... -431 bp Δ5a
B
5’ ···GTTCCGTAGATGCTTTAGACGTTTCCAAA···3’
3’ ···CAAGGCATCTACGAAATCTGCAAAGGTTT···5’
5’ ···AAGCCGAAGACTGGTAGGTTGACTGAAAA···3’
3’ ···TTCGGCTTCTGACCATCCAACTGACTTTT···5’
5b-s1
157bp
5b-s2
F2
R2
CCGTAGATGCTTTAGACGTTTCC(157bp)CCGAAGACTGGTAGGTTGACTGA WT
.....ACTGA -209 bp Δ5b
..... -220 bp Δ5b
CCGT.....TGACTGA -192 bp Δ5b

FIG. S3

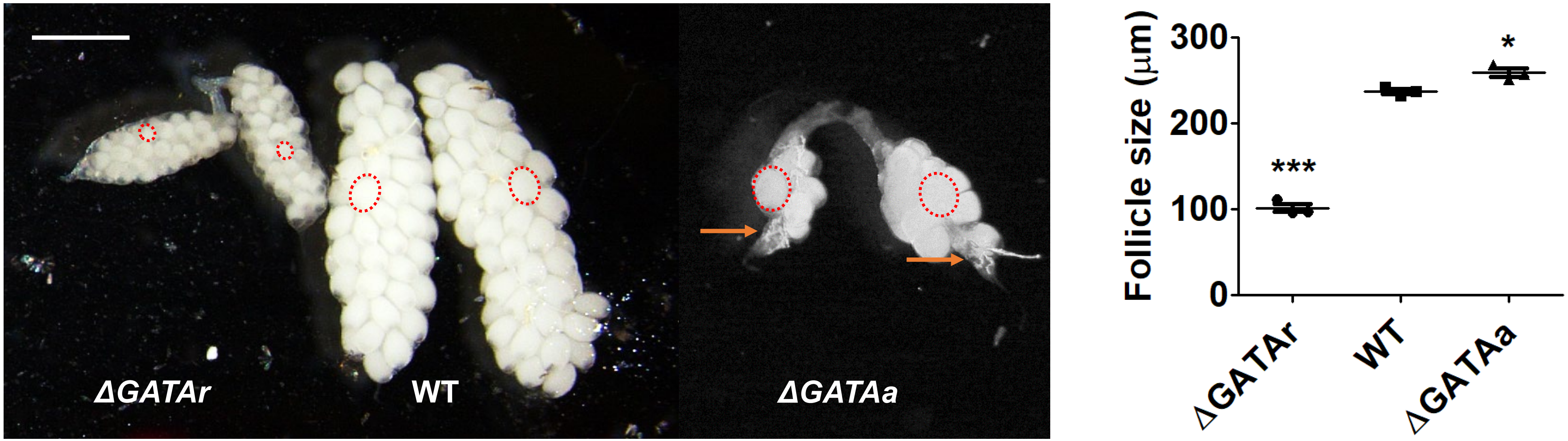
ΔGATAr
WT
ΔGATAa
Follicle size (μm)
300
200
100
0
***
*
ΔGATAr
WT
ΔGATAa

FIG. S4

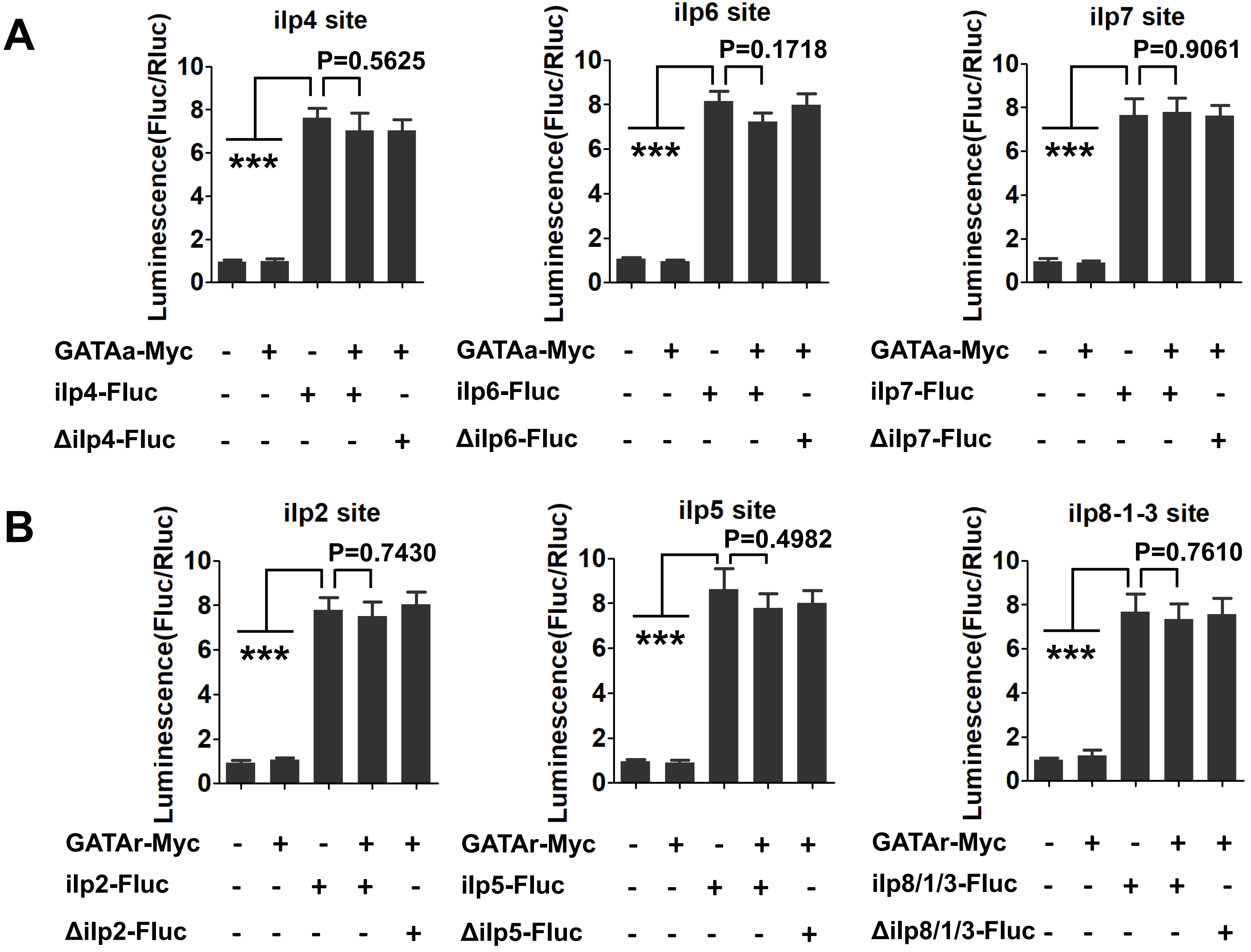
A
ilp4 site
ilp6 site
ilp7 site
Luminescence(Fluc/Rluc)
P=0.5625
P=0.1718
P=0.9061
***
GATAa-Myc - + - + +
ilp4-Fluc - - + + -
Δilp4-Fluc - - - - +
ilp6-Fluc - - + + -
Δilp6-Fluc - - - - +
ilp7-Fluc - - + + -
Δilp7-Fluc - - - - +
B
ilp2 site
ilp5 site
ilp8-1-3 site
P=0.7430
P=0.4982
P=0.7610
GATAr-Myc - + - + +
ilp2-Fluc - - + + -
Δilp2-Fluc - - - - +
ilp5-Fluc - - + + -
Δilp5-Fluc - - - - +
ilp8/1/3-Fluc - - + + -
Δilp8/1/3-Fluc - - - - +

FIG. S5

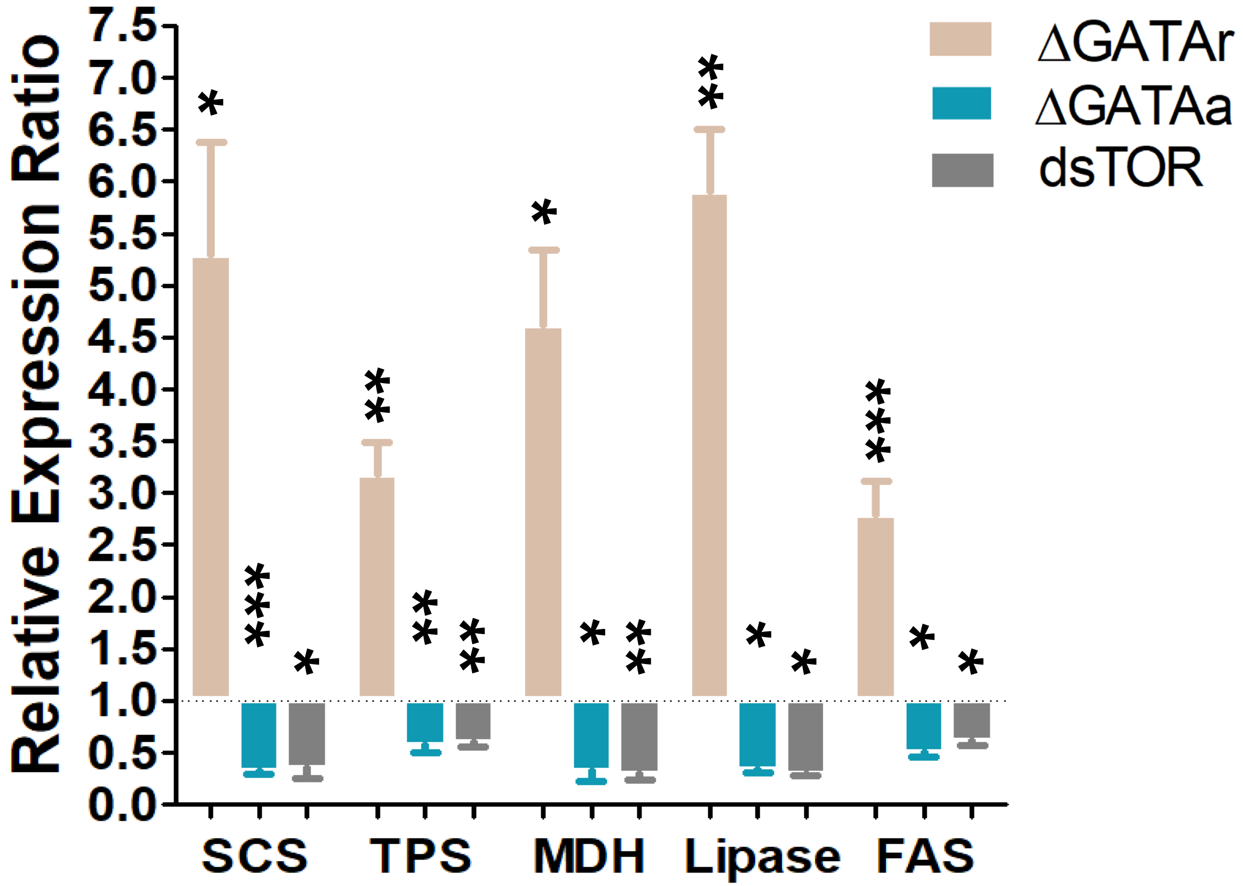

Relative Expression Ratio
7.5
7.0
6.5
6.0
5.5
5.0
4.5
4.0
3.5
3.0
2.5
2.0
1.5
1.0
0.5
0.0
SCS
TPS
MDH
Lipase
FAS
ΔGATAr
ΔGATAa
dsTOR

FIG. S6

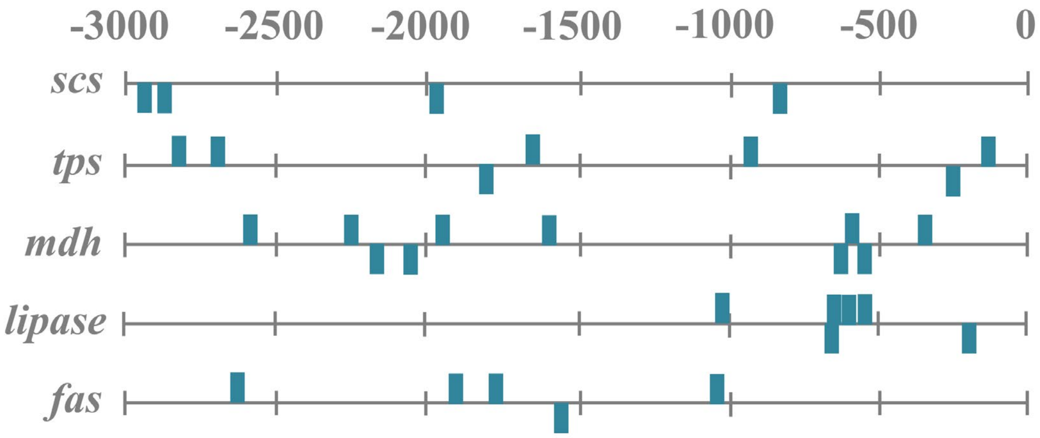
-3000
-2500
-2000
-1500
-1000
-500
0
scs
tps
mdh
lipase
fas

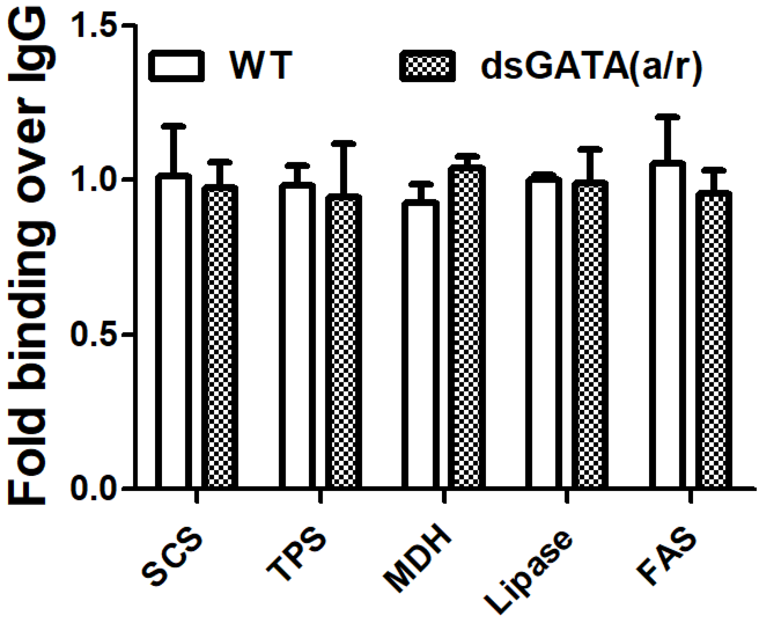
Fold binding over IgG
1.5
1.0
0.5
0.0
WT
dsGATA(a/r)
SCS
TPS
MDH
Lipase
FAS

FIG. S7

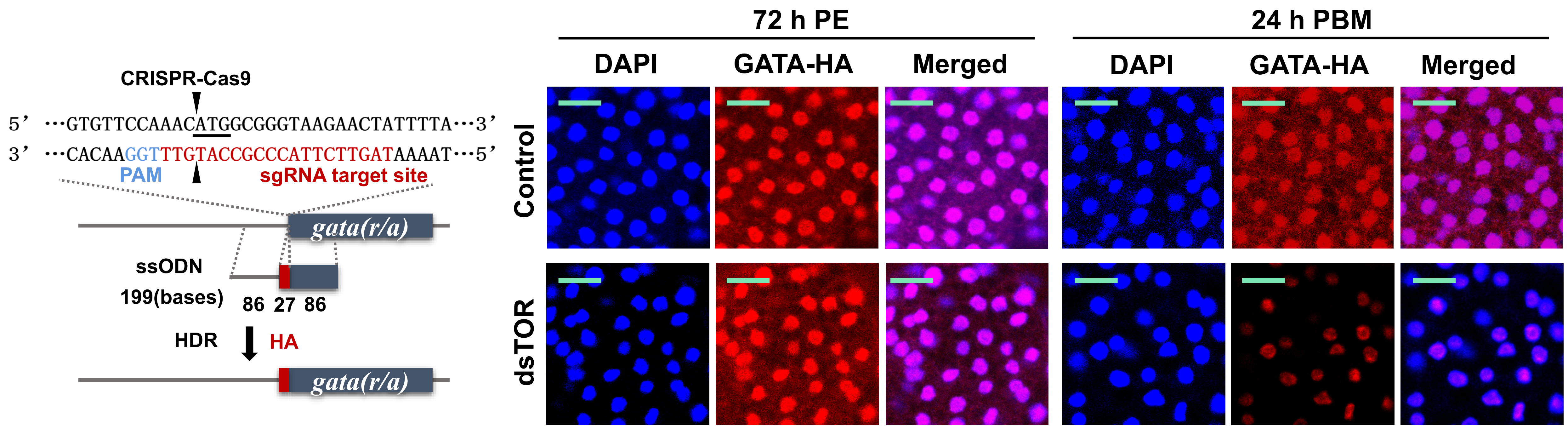
CRISPR-Cas9
5’ ···GTGTTCCAAACATGGCGGGTAAGAACTATTTTA···3’
3’ ···CACAAGGTTTGTACCGCCCATTCTTGATAAAAT···5’
PAM
sgRNA target site
gata(r/a)
ssODN
199(bases)
86 27 86
HDR
HA
gata(r/a)
72 h PE
24 h PBM
DAPI
GATA-HA
Merged
DAPI
GATA-HA
Merged
Control
dsTOR

FIG. S8

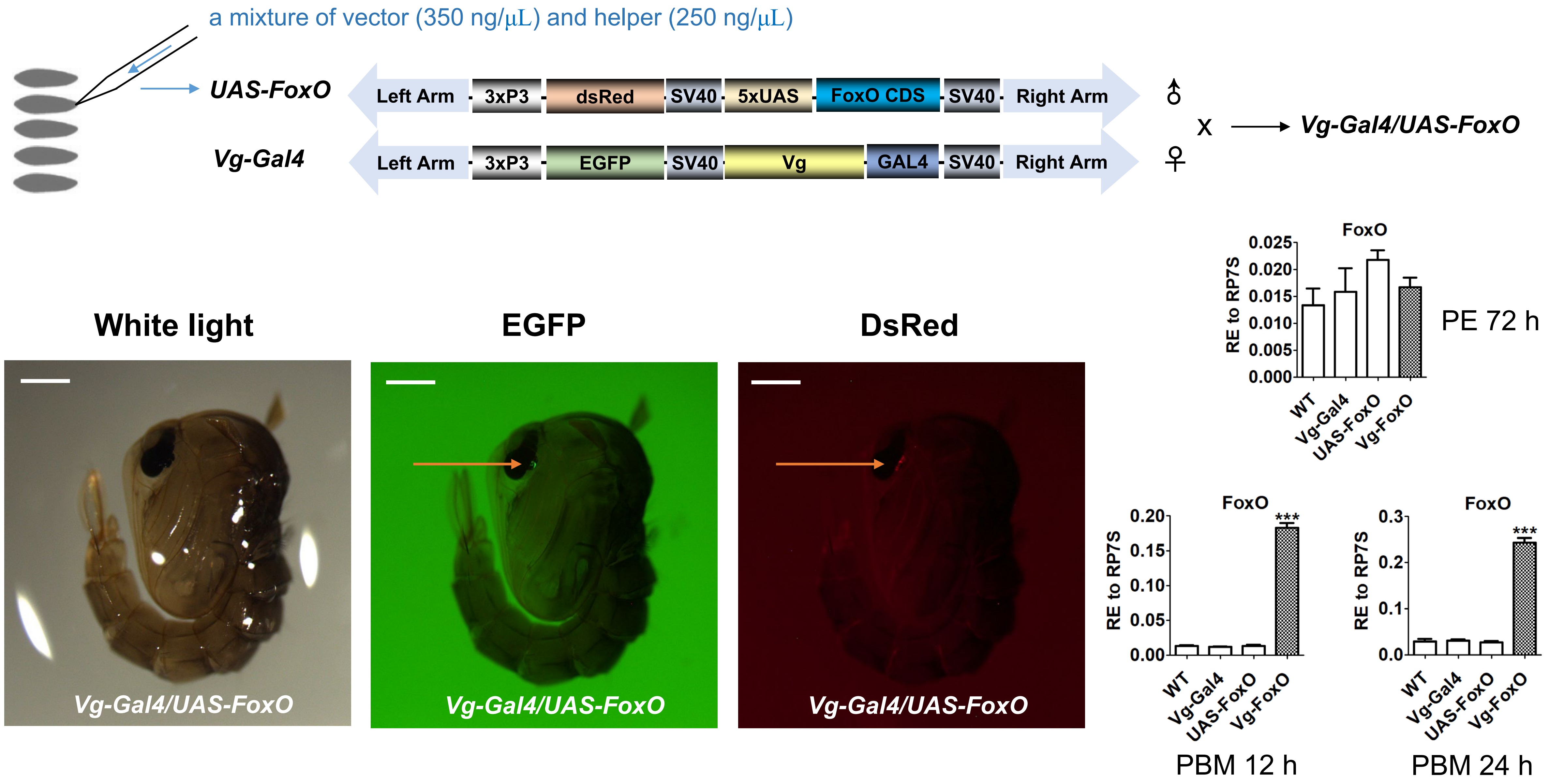

a mixture of vector (350 ng/μL) and helper (250 ng/μL)
UAS-FoxO
Left Arm 3xP3 dsRed SV40 5xUAS FoxO CDS SV40 Right Arm
♂
Vg-Gal4
Left Arm 3xP3 EGFP SV40 Vg GAL4 SV40 Right Arm
♀
x
Vg-Gal4/UAS-FoxO
White light
EGFP
DsRed
Vg-Gal4/UAS-FoxO
Vg-Gal4/UAS-FoxO
Vg-Gal4/UAS-FoxO
FoxO
RE to RP7S
0.025
0.020
0.015
0.010
0.005
0.000
WT
Vg-Gal4
UAS-FoxO
Vg-FoxO
PE 72 h
FoxO
RE to RP7S
0.20
0.15
0.10
0.05
0.00
***
WT
Vg-Gal4
UAS-FoxO
Vg-FoxO
PBM 12 h
FoxO
RE to RP7S
0.3
0.2
0.1
0.0
***
WT
Vg-Gal4
UAS-FoxO
Vg-FoxO
PBM 24 h

FIG. S9

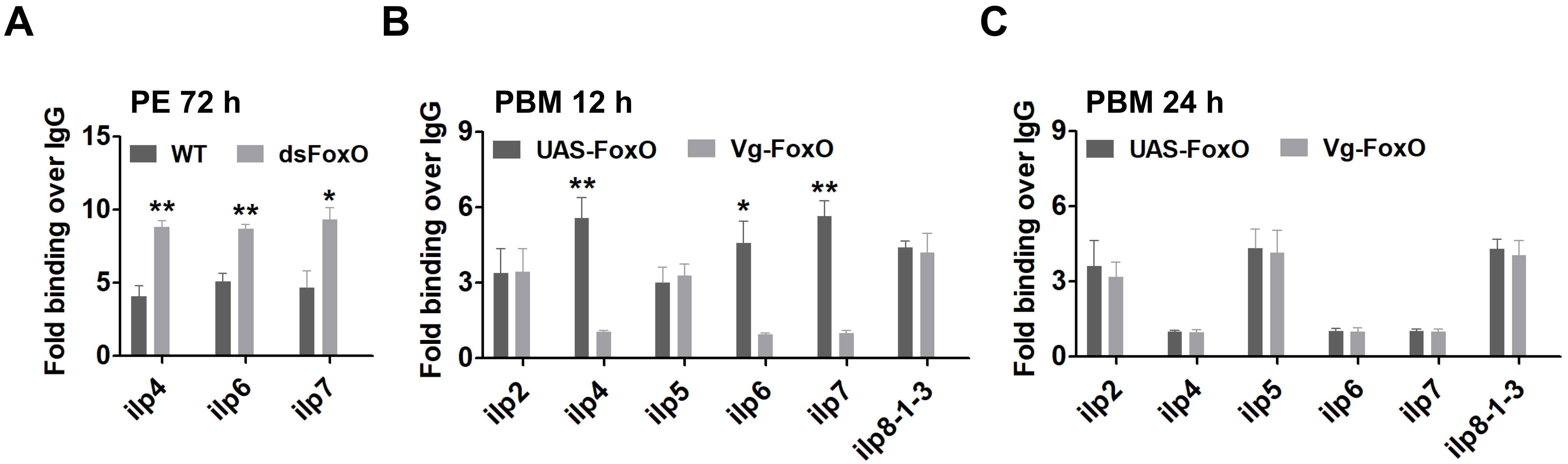
A
B
C
PE 72 h
WT
dsFoxO
Fold binding over IgG
15
10
5
0
**
**
*
ilp4
ilp6
ilp7
PBM 12 h
UAS-FoxO
Vg-FoxO
9
6
3
0
**
*
**
ilp2
ilp4
ilp5
ilp6
ilp7
ilp8-1-3
PBM 24 h
UAS-FoxO
Vg-FoxO
9
6
3
0
ilp2
ilp4
ilp5
ilp6
ilp7
ilp8-1-3
